# Diversity, Biogeography, and Biodegradation Potential of Actinobacteria in the Deep-Sea Sediments along the Southwest Indian Ridge

**DOI:** 10.3389/fmicb.2016.01340

**Published:** 2016-08-29

**Authors:** Ping Chen, Limin Zhang, Xiaoxuan Guo, Xin Dai, Li Liu, Lijun Xi, Jian Wang, Lei Song, Yuezhu Wang, Yaxin Zhu, Li Huang, Ying Huang

**Affiliations:** ^1^State Key Laboratory of Microbial Resources, Institute of Microbiology, Chinese Academy of SciencesBeijing, China; ^2^College of Life Sciences, University of Chinese Academy of SciencesBeijing, China; ^3^Information Network Center, Institute of Microbiology, Chinese Academy of SciencesBeijing, China; ^4^China General Microbiological Culture Collection Center, Institute of Microbiology, Chinese Academy of SciencesBeijing, China; ^5^Shanghai-MOST Key Laboratory of Disease and Health Genomics, Chinese National Human Genome Center at ShanghaiShanghai, China

**Keywords:** community structure, cultivation, biodegradation, marine actinobacteria, microbial diversity, pyrosequencing, Southwest Indian Ridge

## Abstract

The phylum *Actinobacteria* has been reported to be common or even abundant in deep marine sediments, however, knowledge about the diversity, distribution, and function of actinobacteria is limited. In this study, actinobacterial diversity in the deep sea along the Southwest Indian Ridge (SWIR) was investigated using both 16S rRNA gene pyrosequencing and culture-based methods. The samples were collected at depths of 1662–4000 m below water surface. Actinobacterial sequences represented 1.2–9.1% of all microbial 16S rRNA gene amplicon sequences in each sample. A total of 5 actinobacterial classes, 17 orders, 28 families, and 52 genera were detected by pyrosequencing, dominated by the classes *Acidimicrobiia* and *Actinobacteria*. Differences in actinobacterial community compositions were found among the samples. The community structure showed significant correlations to geochemical factors, notably pH, calcium, total organic carbon, total phosphorus, and total nitrogen, rather than to spatial distance at the scale of the investigation. In addition, 176 strains of the *Actinobacteria* class, belonging to 9 known orders, 18 families, and 29 genera, were isolated. Among these cultivated taxa, 8 orders, 13 families, and 15 genera were also recovered by pyrosequencing. At a 97% 16S rRNA gene sequence similarity, the pyrosequencing data encompassed 77.3% of the isolates but the isolates represented only 10.3% of the actinobacterial reads. Phylogenetic analysis of all the representative actinobacterial sequences and isolates indicated that at least four new orders within the phylum *Actinobacteria* were detected by pyrosequencing. More than half of the isolates spanning 23 genera and all samples demonstrated activity in the degradation of refractory organics, including polycyclic aromatic hydrocarbons and polysaccharides, suggesting their potential ecological functions and biotechnological applications for carbon recycling.

## Introduction

The phylum *Actinobacteria* is composed of a large group of morphologically and physiologically diverse Gram-positive bacteria with high genomic G+C contents, which are ubiquitous in nature (Ensign, 1992; Goodfellow et al., 2012). Members of this phylum are also successful colonizers of different extreme environments, often occurring as abundant populations (Bull, 2011). This phylum has been found to be one of the top five most abundant bacterial phyla in the deep ocean (Zinger et al., 2011; Yilmaz et al., 2016).

The marine actinobacterial diversity has been mainly investigated by culture-dependent methods, and at least 66 actinobacterial genera have been cultured from different marine environments (Goodfellow and Fiedler, 2010; http://www.bacterio.net/). However, limited efforts have been devoted to exploring actinobacterial diversity and community compositions in the deep sea, particularly by using high-throughput sequencing approaches. A recent investigation using clone sequencing detected 9 actinobacterial genera from deep Arctic marine surface sediments (Zhang et al., 2014); and a targeted search for the marine actinomycetes *Salinispora* spp. using specific 16S rRNA gene primers revealed the presence of this genus in deep marine sediments (3814–5699 m below water surface and 0–7 m below see floor [mbsf]) from the Canary Basin and South Pacific Gyre (Prieto-Davó et al., 2013). Data from previous studies on deep-sea microbial diversity showed that the relative abundance of actinobacteria in the sediments were 0.1−3.0% in the Arctic Ocean (0.38–2.32 mbsf; Jorgensen et al., 2012), 0.7−2.4% in the Atlantic Ocean (0–0.02 mbsf; Schauer et al., 2010), and about 10.0% in the Pacific Ocean (0–350 mbsf; Inagaki et al., 2006). In the hydrothermal surface sediments, the relative abundance of actinobacteria was found to be about 5.0% at the Mid-Atlantic Ridge (López-García et al., 2003) and about 3.8% in the Guaymas Basin of Pacific Ocean (Teske et al., 2002). The metatranscriptomes of anaerobic Peru Margin sediment from 5 to 159 mbsf also indicated relatively abundant gene expression from *Actinobacteria* (1.2–6.1%; Orsi et al., 2013). As for planktonic actinobacteria in the ocean, it was shown that their global distribution changed along latitudinal gradients (Pommier et al., 2007); however, their composition in the brackish northern Baltic Sea was related to environmental gradients including total phosphorus (TP), dissolved organic carbon, chlorophyll a, and bacterial production (Holmfeldt et al., 2009). These findings suggest a biogeographic distribution of actinobacteria that is shaped by biogeochemical parameters such as dissolved organic carbon, TP, etc. However, little is known about the actinobacterial diversity and biogeography in deep-sea habitats.

The Southwest Indian Ridge (SWIR) is one of the slowest spreading ocean ridges and has diverse geographic environments including an unexpected high frequency of hydrothermal vents and plumes (German et al., 1998; Tao et al., 2012, 2014). Hydrothermal systems are enriched with a variety of chemical substances (such as manganese [Mn], iron [Fe], methane [CH_4_], hydrogen gas [H_2_], reduced sulfur compounds, and polycyclic aromatic hydrocarbons [PAHs]) and form dynamic habitats with steep thermal and chemical gradients that may influence the microbial structure (Jannasch and Mottl, 1985; Baker et al., 1995; Simoneit and Fetzer, 1996). Due to its remote location, however, the SWIR is not readily accessible to investigate (Tao et al., 2012). Recently, a clone library study revealed that *Thaumarchaeota, Acidobacteria, Actinobacteria, Bacteroidetes*, and *Proteobacteria* dominated the archaeal and bacterial communities in a semi-consolidated carbonate sample of the SWIR and also suggested that *Alphaproteobacteria* and *Thaumarchaeota* potentially participated in sulfur (S) and nitrogen cycles in this environment (Li et al., 2014). Supporting this, another study demonstrated that *Alphaproteobacteria, Gammaproteobacteria, Deltaproteobacteria, Firmicutes, Nitrospirae*, and archaea might participate in the S cycle in two low-temperature hydrothermal deposits at the SWIR (Cao et al., 2014). Nevertheless, it remains unclear about the abundance and composition of actinobacteria at the SWIR and the biogeochemical processes that they are involved in.

Besides the well-known ability of actinobacteria to produce diverse bioactive compounds, they also produce various extracellular hydrolytic enzymes, which have important ecological roles and potential biotechnological applications (Peczynska-Czoch and Mordarski, 1988). These enzymes are widely reported among soil actinobacteria and have been shown to participate in the decomposition of recalcitrant organic carbons such as polysaccharides (Manucharova, 2009). Moreover, members of the actinobacteria have also been reported as highly efficient PAH degraders in PAH-contaminated soils (Uyttebroek et al., 2006; García-Díaz et al., 2013). Cellulose, chitin, and pectin are the main components of eukaryotic cell walls, and thus are abundant and widely distributed in marine environments, serving as important sources of organic matter. Many of the actinobacteria isolated from coastal and shallow marine sediments are capable of producing cellulase, chitinase, and pectinase (Veiga et al., 1983; Augustine et al., 2013). However, there are few reports corresponding to such capabilities of deep-sea actinobacteria. PAHs are the main components of petroleum and are introduced into the marine environment by oil spill, river import, natural seepage, hydrothermal activity, and even air current transfer (Latimer and Zheng, 2003). PAHs are persistent organic pollutants that tend to accumulate in marine sediments and have even been reported to be widespread in the deep Arctic Ocean and Pacific Ocean (Simoneit and Fetzer, 1996; Dong et al., 2015). A recent study indicated that hydrocarbon-degrading actinobacteria, notably *Dietzia*, were abundant in the Arctic deep-sea sediments (0–0.5 mbsf) and were likely to play an important role in PAH degradation *in situ* (Dong et al., 2015).

In this study, we explored the diversity of actinobacteria at the SWIR using 16S rRNA gene pyrosequencing and culture-dependent methods. We determined how the actinobacterial community from different sampling sites varied in composition and identified the environmental factors that contributed to the distribution of actinobacteria. The degradation capacity of diverse actinobacterial isolates toward recalcitrant organic substances was also evaluated, which might help to uncover their ecological functions and biotechnological applications. Our results provide new insights into the actinobacterial community structure at the SWIR and contribute to the understanding of the underlying mechanisms behind actinobacterial distribution in the deep ocean.

## Materials and methods

### Sample collection and physicochemical analysis

**Figure 1:**
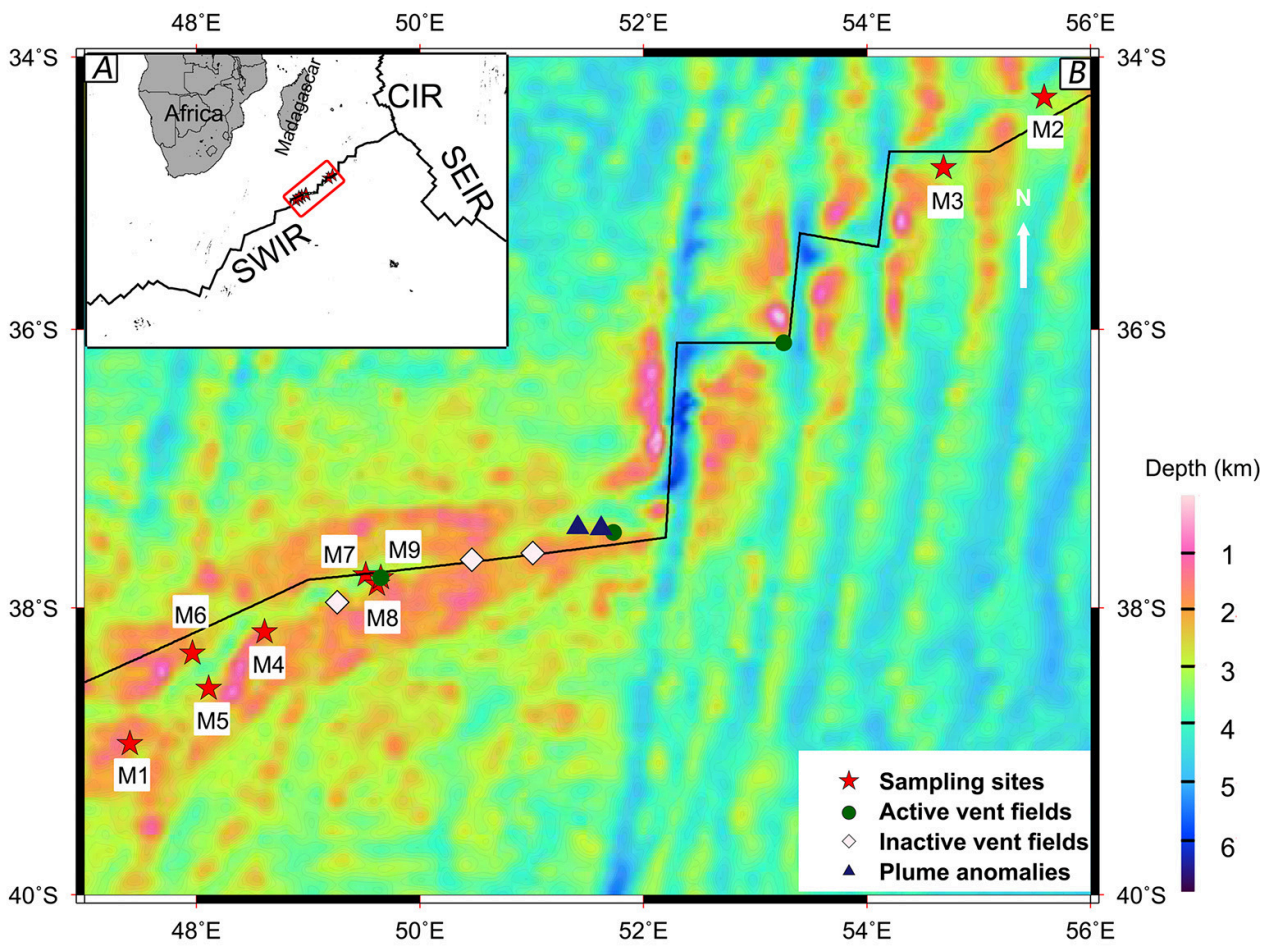
**The map of sampling sites along the Southwest Indian Ridge (SWIR)**. The hydrothermal field information was collected from previous studies (Tao et al., 2012, 2014). **(A)** Location of the SWIR, near the Southeast Indian Ridge (SEIR) and the Central Indian Ridge (CIR). **(B)** Magnified view of the sampling area marked by a red box in A using a bathymetric map.

Eight surface sediment samples (M1–M8) and a bottom water sample (M9) were collected along the SWIR at depths ranging from 1662 to 4000 m during the DY115-21 and DY115-22 cruises of the DaYang YiHao research vessel in January 2010, March 2010, and January 2011 (Figure 1, Table 1). Samples M8 and M9 were collected from the first discovered active hydrothermal field at the SWIR (Tao et al., 2012). A television multi-core sampler (Φ10 × 60 cm) was deployed to collect surface sediment samples and a rosette water sampler equipped with 10-l Niskin bottles and a CTD unit was used to collect the water sample. The sediment samples (0–0.2 mbsf) were transferred from the cores into sterile 200-ml plastic boxes using sterile scoops. Water for physicochemical analysis was collected in a sterile 1-l glass bottle. Microbial cells were collected from 50 l of water by a tangential flow filtration device (Millipore, Bedford, MA, USA) and further concentrated by subsequent filtration onto two polycarbonate filters (diameter 47 mm, pore size 0.1 μm, Millipore) in the onboard laboratory. All samples were frozen at −20°C until further processing in the laboratory.

**Table 1:** Environmental parameters of the samples^*^.

**Parameter^†^**	**M1**	**M2**	**M3**	**M4**	**M5**	**M6**	**M7**	**M8**	**M9**
Sample property	Sediment	Sediment	Sediment	Sediment	Sediment	Sediment	Sediment	Sediment	Seawater
Sampling date	16 March 2010	24 March 2010	25 March 2010	08 January 2011	08 January 2011	09 January 2011	13 January 2010	15 January 2010	11 January 2010
Longitude	47.4069°E	55.5838°E	54.6835°E	48.6123°E	48.1095°E	47.9646°E	49.5167°E	49.6167°E	49.6500°E
Latitude	38.9533°S	34.2985°S	34.8192°S	38.1668°S	38.5650°S	38.3187°S	37.7667°S	37.8333°S	37.7833°S
Depth (m)	1880	3178	3523	2187	2734	1662	4000	2800	2800
EC (us/cm)	6500	12040	9920	10160	10740	7780	4170	11520	26200
pH	9.06	8.83	8.62	9.11	8.96	8.9	8.34	7.77	7.47
Ca (g/kg)	290.30	340.10	353.30	299.50	337.90	290.60	20.01	0.15	3.19
Fe (mg/kg)	2610.02	4229.17	2982.88	2159.43	3902.49	8666.48	364.74	1553.71	0.02
Mg (mg/kg)	8300.00	2900.00	1800.00	1200.00	2000.00	13300.00	158.00	62.58	9312.00
Mn (mg/kg)	1428.70	596.23	394.15	277.52	551.21	12915.74	19.19	105.02	0.01
S (mg/kg)	2067.02	2036.25	1800.20	1218.69	966.65	1133.50	300.37	44.46	1254.50
TOC (mg/kg)	1300.00	1600.00	1400.00	1700.00	1400.00	1300.00	4920.00	68255.00	18.01
TN (mg/kg)	740.00	390.00	530.00	870.00	550.00	790.00	389.00	239.00	47.00
NO-N (mg/kg)	57.00	19.00	19.00	23.00	20.00	27.00	13.91	5.12	8.84
TP (mg/kg)	170.00	370.00	230.00	150.00	180.00	210.00	1590.00	2590.00	679.00

* *The temperature at all sampling locations was 2–4°C*.

† *EC, electric conductance; TN, total nitrogen; TP, total phosphorus; TOC, total organic carbon; NO-N, nitrate nitrogen*.

Sample geochemical analyses were conducted at the Analysis Center of Tsinghua University. The samples were mixed with distilled water without CO_2_ (Millipore) at a 1:2.5 ratio, and the electric conductance and pH of the mixtures were determined using a pH/conductivity meter (HACH Sension 156). The concentrations of total organic carbon (TOC) and total nitrogen (TN) were measured using the combustion method with the Elemental Analyzer (Euroea, Eurouector, Italy). Major elements Mg, S, Ca, Mn, and Fe were analyzed using inductively coupled plasma atomic emission spectroscopy (ICP-AES) (VISTA-MPX, VARIN, USA). The concentration of TP was determined using the modified Murphy Riley method (Carter and Gregorich, 2008). Nitrate nitrogen (NO-N) was quantified using the automated spectrophotometric method (Carter and Gregorich, 2008). The longitude, latitude, depth, and temperature were recorded by the research vessel. Detailed information of the samples is provided in Table 1. The map of sampling sites was drawn using the Generic Mapping Tools (Wessel and Smith, 1998) (http://gmt.soest.hawaii.edu/). Mercator projection and NUVEL-1A plate boundaries (http://jules.unavco.org/GMT/) were used for plotting.

### DNA extraction, PCR amplification and pyrosequencing of the 16S rRNA gene

Filters used to collect cells from the water samples were smashed in 20 ml phosphate buffered solution in an ice bath using the Tissue Tearor (Model: 985370-395, Biospec Products, INC). A sample of sediment (5 g [wet weight]) or filter homogenate (5 ml) was subjected to DNA extraction using the high-salt-SDS-heat method (Zhou et al., 1996), with a modification that the mixture of sample and extraction buffer was frozen in liquid nitrogen and thawed in a 65°C water bath for three cycles and then cooled down to 37°C before proteinase K was added. For each sample, three replicates of DNA extracts were obtained and pooled together for the subsequent PCR amplication and pyrosequencing.

Due to the low coverage and relatively high false positive rate of the reported *Actinobacteria*-specific 16S rRNA gene primers (Stach et al., 2003b; Schäfer et al., 2010) and in order to investigate the actinobacterial relative abundance among the prokaryotic community, a pair of universal 16S rRNA gene primers was used in the present study. The total DNA was amplified in triplicate by PCR for pyrosequencing, using the primers U789F (5′-TAGATACCCSSGTAGTCC-3′) and U1068R (5′-CTGACGRCRGCCATGC-3′; Baker et al., 2003; Lee et al., 2010) with 8-nucleotide barcodes. These primers target the V5–V6 region on the 16S rRNA gene and cover about 93.0–96.0% of bacteria and archaea (Klindworth et al., 2012). PCR was performed in triplicate in 50-μl reaction system containing 5U of *Pfu* DNA polymerase, 1 × *Pfu* reaction buffer, 0.2 mM of dNTPs (TaKaRa, Japan), 0.4 μM of each barcoded primers, and 100 ng of genomic DNA template. The amplification was conducted under the following conditions: initial denaturation at 94°C for 5 min; 26 cycles of denaturation at 94°C for 30 s, annealing at 53°C for 30 s and extension at 72°C for 45 s; and a final extension at 72°C for 6 min (Lee et al., 2010). The triplicate PCR products were pooled and purified using the TaKaRa Agarose Gel DNA Purification Kit (Takara, Japan). The amplicons from nine samples were quantified by Qubit dsDNA HS Assay Kit (Life Technologies, Eugene, Oregon, USA) on the Qubit 2.0 Fluorometer (Invitrogen, Carlsbad, CA, USA) and then mixed in equal amounts. The prepared DNAs were transformed into single-stranded template DNA (sstDNA) libraries by using the GS DNA Library Preparation Kit (Roche Applied Science). The sstDNA libraries were clonally amplified in a bead-immobilized form by using the GS emPCR Kit (Roche Applied Science) and then sequenced on the 454 Genome Sequencer GS FLX+ Titanium Platform (Roche Applied Science) at the Chinese National Human Genome Center, Shanghai, China.

### Pyrosequencing data processing and statistical analyses

Pyrosequencing data were processed using Quantitative Insights Into Microbial Ecology (QIIME 1.7; Caporaso et al., 2010b). Sequences that contained ambiguous bases or were shorter than 150 bp in length were discarded; only those with complete barcodes that were 100% identical to the expected barcodes, no more than three mismatches in the primer sequences (Huse et al., 2007), and an average quality score >20 were included in further analyses. Prior to the analysis, chimeric sequences were detected and excluded from the de-noised sequences using the QIIME implementation of the ChimeraSlayer algorithm (DeSantis et al., 2006; Haas et al., 2011). The high quality sequences were deposited in the NCBI database under accession number SRP046759. Uclust was used to identify phylotypes and assign sequences to operational taxonomic units (OTUs) at a distance cutoff of 0.03 (Edgar, 2010). The cluster seeds of OTUs were chosen as the representative sequences and aligned using PyNAST (Caporaso et al., 2010a) with the greengenes core set (DeSantis et al., 2006) as the template. The phylogenetic tree of the representative sequences was constructed using FastTree (Price et al., 2010). The software RDP classifier (Wang et al., 2007) was used to assign sequences to phylogenetic taxa based on the Ribosomal Database Project (Cole et al., 2013) under the condition of a bootstrap cutoff of 50%. Actinobacterial reads unclassified at the family level were further annotated by online SINA 1.2.11 (Pruesse et al., 2012) based on the SILVA SSU Ref NR 99 123.1 database. The alpha_diversity.py QIIME script was also used to calculate Shannon diversity estimators and Simpson's diversity index after rarefying reads to an even depth (*n* = 108 reads) across samples.

The dissimilarity of phylogenetic diversity among the actinobacterial communities was measured by a weighted UniFrac distance matrix (Hamady et al., 2010). To correct the sampling error, the samples were rarefied to the minimal number of sequences for actinobacterial community. Dissimilarity matrices for geochemical characteristics were calculated using Euclidean distances, after standardizing the data to *Z*-score to make them comparable. A geographic distance matrix was calculated using the Vincenty formula and ln transformed for the correlation analysis as suggested by Martiny et al. (2011; Table S1). The dissimilarities of actinobacterial community compositions and environmental variables among samples were evaluated using the principal coordinate analysis (PCoA) method in QIIME, with weighted UniFrac and Euclidean distances, respectively. The Mantel test was used to assess the correlations between the actinobacterial community dissimilarity and environmental variables by 99,999 permutations in QIIME. Detrended correspondence analysis (Hill and Gauch, 1980), canonical correspondence analysis (Ter Braak, 1986), and partial canonical correspondence analysis (Borcard et al., 1992) were also performed to compute the correlations between the community structure and environmental variables in R version 3.1.0 (R Core Team, 2014) using the functions in the Vegan package (Oksanen et al., 2013). Environmental variables with variance inflation factors over 20, such as Ca and Mn, were removed from the canonical correspondence analysis in order to exclude multicollinearity in the model (Yang et al., 2014).

### Selective isolation of actinobacteria

The samples were processed using the following three methods.

- Ultrasonication/dilution (Qiu et al., 2008). One gram of sediment or 1 ml of filter homogenate were added to 4 ml of sterile artificial seawater (distilled water containing 3.3% sea salts), followed by shaking for 2 h at 28°C and 180 rpm and processing in a water bath sonicator (model KQ-100DB, 40 kHz, 100 W; Kunshan Ultrasonic instruments Co., Ltd., Kunshan, China) for 2 min at 30°C. The resulting samples were further diluted (1:1) with sterile artificial seawater.
- Dispersion and differential centrifugation technique (Hopkins et al., 1991). One gram of sediment or 1 ml of filter homogenate were blended with 5 ml 0.1% (w/v) sodium cholate, 5 ml chelating resin (Na^+^, Sigma), and approximately 10 glass beads, shaken for 2 h at 10°C, and centrifuged at 500 g for 2 min to get the supernatant. The residue was resuspended in 5 ml Tris buffer (pH 7.4), shaken for 1 h at 10°C, and centrifuged at 500 g for 1 min to get the supernatant. The two supernatants were pooled together as supernatant 1. The above residue was resuspended in 5 ml sodium cholate and processed in an ultrasonic bath for 1 min, mixed with 5 ml sodium cholate and shaken for 1 h at 10°C, and then centrifuged at 500 g for 1 min to get supernatant 2. The resulting residue was resuspended in 10 ml sterile distilled water, shaken for 1 h at 10°C, and centrifuged at 500 g for 2 min to get supernatant 3. The three supernatants of each sample were combined and centrifuged at 5000 g for 20 min, and then the residue was resuspended in 1 ml sterile artificial seawater for subsequent inoculation.
- Enrichment/dilution. The ultrasonication/dilution sample was inoculated (1:10) in duplicate into SMP (Jensen et al., 2005) and M5 (Zhang et al., 2006) media without agar in 15-ml centrifuge tubes, followed by incubation for 8 weeks at 16°C with the addition of fresh antibiotics (50 mg l^−1^ each of nalidixic acid and cycloheximide) every 2 weeks.

The pretreated samples were then diluted by 10 and 100 in sterile artificial seawater. Amounts of 100 μl of each of the resulting dilutions were inoculated in quadruplicate onto the surface of the following selective agar media: AMM, SMP, SNC, and SRC (Jensen et al., 2005), 1/10 AMM, ISP 2 (modified with 0.2% CaCO_3_), ISP 3 (Shirling and Gottlieb, 1966), and M5. All media were prepared with either natural seawater or artificial seawater and supplemented with 0.1% (v/v) vitamin solution (Janssen et al., 1997), 0.1% (v/v) trace salt solution (Shirling and Gottlieb, 1966), and 50 mg l^−1^ each of nalidixic acid and the anti-fungal agent cycloheximide or nystatin. The initial pH of each medium was adjusted to 7.2. The quadruplicate plates were divided into two sets and incubated respectively at 28 and 10°C for 2–15 weeks. The plates were monitored every week and almost all growing colonies were transferred onto new AMM plates until pure cultures were obtained. The pure isolates were cryopreserved in 20% glycerol at −80°C.

### 16S rRNA gene sequencing of the isolates and phylogenetic analysis

Genomic DNA extraction from the isolates was performed as previously described (Chun and Goodfellow, 1995). Nearly complete 16S rRNA genes were PCR amplified using the universal primers 27f and 1492r (Lane, 1991). Reaction products were checked, purified, and directly sequenced using the previously described method (Guo et al., 2008). Nearly full-length 16S rRNA gene sequences were obtained and edited with MEGA 5.20 (Tamura et al., 2011). The nearest related taxa were retrieved from the GenBank non-redundant database using a BLASTN search (http://www.ncbi.nlm.nih.gov/). Phylogenetic analysis of the isolates was conducted with MEGA 5.20. The phylogenetic tree was constructed using the maximum likelihood (ML) algorithm (Felsenstein, 1981) and the tree topology was evaluated by bootstrap analysis (Felsenstein, 1985) based on 1000 resamplings. The phylogenetic relationships of the isolates and actinobacterial sequences from the pyrosequencing data set were also analyzed. The alignment of all the isolate sequences and representative sequences of actinobacterial OTUs was performed using SINA (SILVA Incremental Aligner) web-based tool (Pruesse et al., 2012). ML phylogenetic tree reconstruction was performed with RAxML version 7.3.0 (Felsenstein, 1981; Stamatakis, 2006) with 100 rapid bootstrap inferences. The jModeltest 2.1 (Darriba et al., 2012) was used to select the best-fit substitution model in the above phylogenetic analyses, which was General Time Reversible model with Gamma distribution and Invariable sites (GTR+G+I). Phylogenetic trees were edited with MEGA 5.20 (Tamura et al., 2011).

The 16S rRNA gene sequences of the actinobacterial isolates were deposited in the GenBank database with accession numbers KM507587–KM507721, HQ622494–HQ622502, HQ622504–HQ622509, HQ622514–HQ622528, HQ622530–HQ622532, JF346424–JF346428, JF346418, JF346420, and JF346422.

### Biodegradation of recalcitrant organic matter

All actinobacterial isolates were qualitatively screened for their activity in the degradation of cellulose, chitin, pectin, fluoranthene, and phenanthrene using the previously described media and methods with modifications (Kiyohara et al., 1982; Veiga et al., 1983; Augustine et al., 2013). Briefly, each actinobacterial strain was spot inoculated in triplicate on nutrient agar containing sodium carboxymethyl cellulose (1%), colloidal chitin (0.2%), pectin (0.5%), fluoranthene (10 mg l^−1^), and phenanthrene (10 mg l^−1^), respectively. Plates were incubated for 3–15 days at 28°C, and then colonies with transparent zones were considered as recalcitrant organic matter-degrading isolates. The cellulose and pectin plates were stained for 10 min with 0.5% congo red and then bleached for 10–20 min with 1 M NaCl before the observation of transparent zones.

## Results

### Environmental characteristics of the study sites

**Figure 2:**
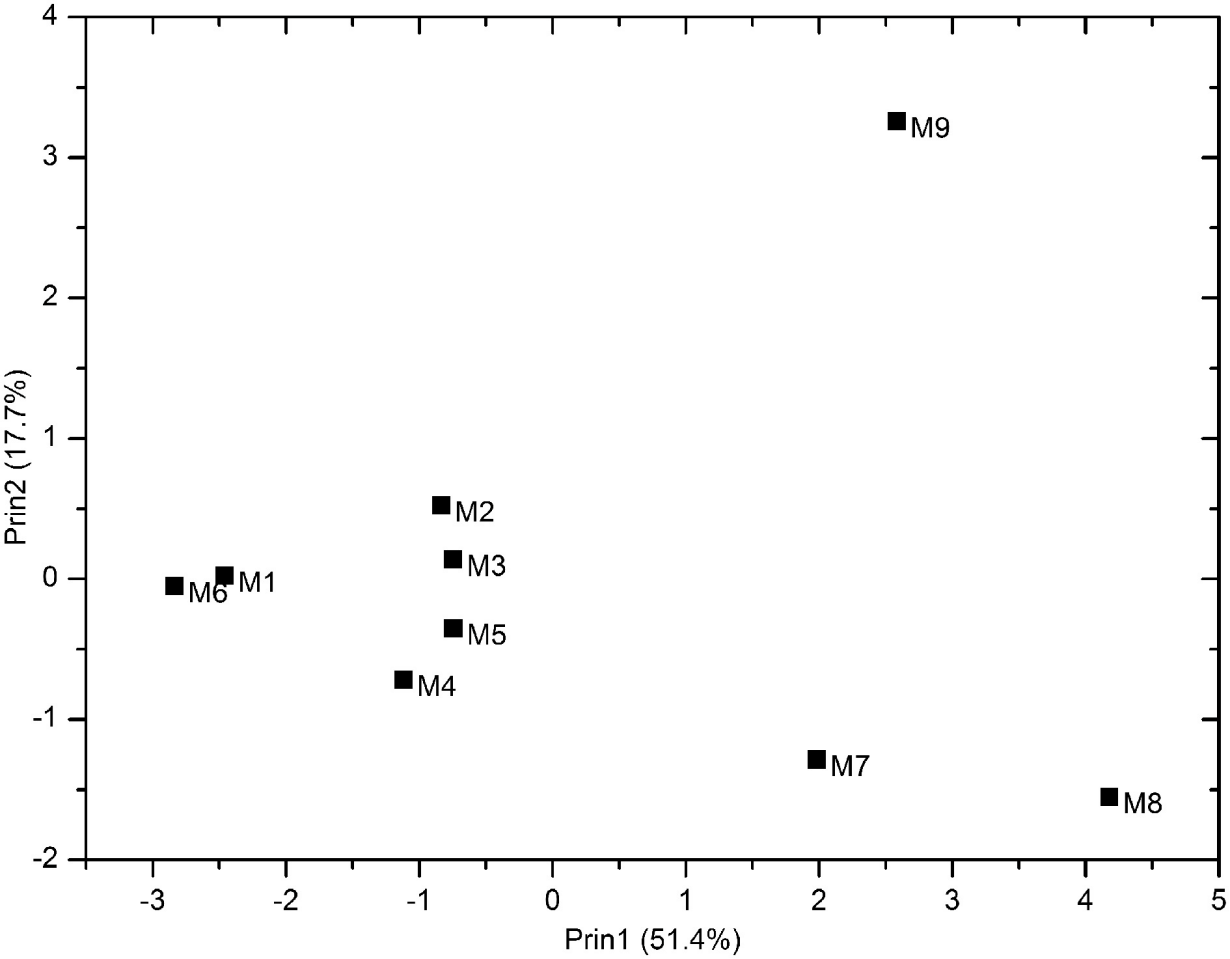
**PCoA representing the similarity of 11 physicochemical factors of the samples**.

Eleven physicochemical and three geographic parameters of the samples were obtained (Table 1). PCoA analysis of the *Z*-scored physicochemical factors showed that samples collected from different sites varied from one another, and samples M8 and M9, which were collected from a hydrothermal field, were distinct from the others (Figure 2). The pairwise geographic distance between the sampling sites ranged from 5.2 (between M8 and M9) to 964.9 km (between M1 and M2; Figure 1; Table S1).

### Community compositions of *Actinobacteria*

A total of 72,889 quality-filtered 16S rRNA gene sequences with an average read length of approximately 250 bp were obtained for further analysis, with 49,717 sequences belonging to bacteria and 23,057 to archaea. Altogether, 28 bacterial and two archaeal phyla were recovered. Among them, *Proteobacteria, Thaumarchaeota, Actinobacteria, Planctomycetes*, and *Firmicutes* were the most abundant (relative abundance >1% in each sample). The phylum *Actinobacteria* represented 4.5% (3245 sequences) of the total reads and 1.2–9.1% of the reads among the samples. The actinobacterial sequences could be divided into 764 OTUs using Uclust at a distance of 0.03 and each sample contained 58–219 OTUs. None of the actinobacterial OTUs were distributed in all samples. The actinobacterial α-diversity, based on the Shannon and Simpson diversity indexes, was not significantly different between the samples (Table 2).

**Table 2:** **The number of OTUs and the estimators of richness and diversity at 0.03 dissimilarity**.

**Sample ID**	**Total reads**	**Total OTUs**	**Total coverage**	**Actinobacterial reads (%)**	**Actinobacterial OTUs (%)**	Shannon^*^	Simpson^*^
M1	7436	3548	0.65	293 (3.9)	130 (3.7)	5.34	0.97
M2	8079	3955	0.64	108 (1.3)	64 (1.6)	5.17	0.96
M3	7500	3559	0.65	136 (1.8)	65 (1.8)	5.13	0.95
M4	6442	3295	0.62	351 (5.4)	118 (3.6)	4.30	0.87
M5	11060	4794	0.68	774 (7.0)	193 (4.0)	4.51	0.93
M6	9807	3897	0.71	119 (1.2)	58 (1.5)	5.09	0.96
M7	6770	2848	0.68	617 (9.1)	171 (6.0)	4.90	0.95
M8	8826	3535	0.73	274 (3.1)	72 (2.0)	3.98	0.89
M9	6969	2493	0.74	573 (8.2)	219 (8.8)	5.05	0.94
Total	72889	23836	0.73	3245 (4.5)	764 (3.2)		

* *Index for actinobacterial community*.

Five known actinobacterial classes (*Acidimicrobiia, Actinobacteria, Coriobacteriia, Rubrobacteria*, and *Thermoleophilia*) were detected, of which *Actinobacteria* and *Acidimicrobiia* were the most abundant (Figure S1). A total of 300 OTUs spanning nearly half (47.7%) of the actinobacterial reads could not be assigned to any known classes within the taxonomic framework of the phylum *Actinobacteria* when using the RDP classifier. These OTUs accounted for 42.0–74.1% of the actinobacterial reads in samples M1–M7 while only 5.8% in M8 and 6.6% in M9.

**Figure 3:**
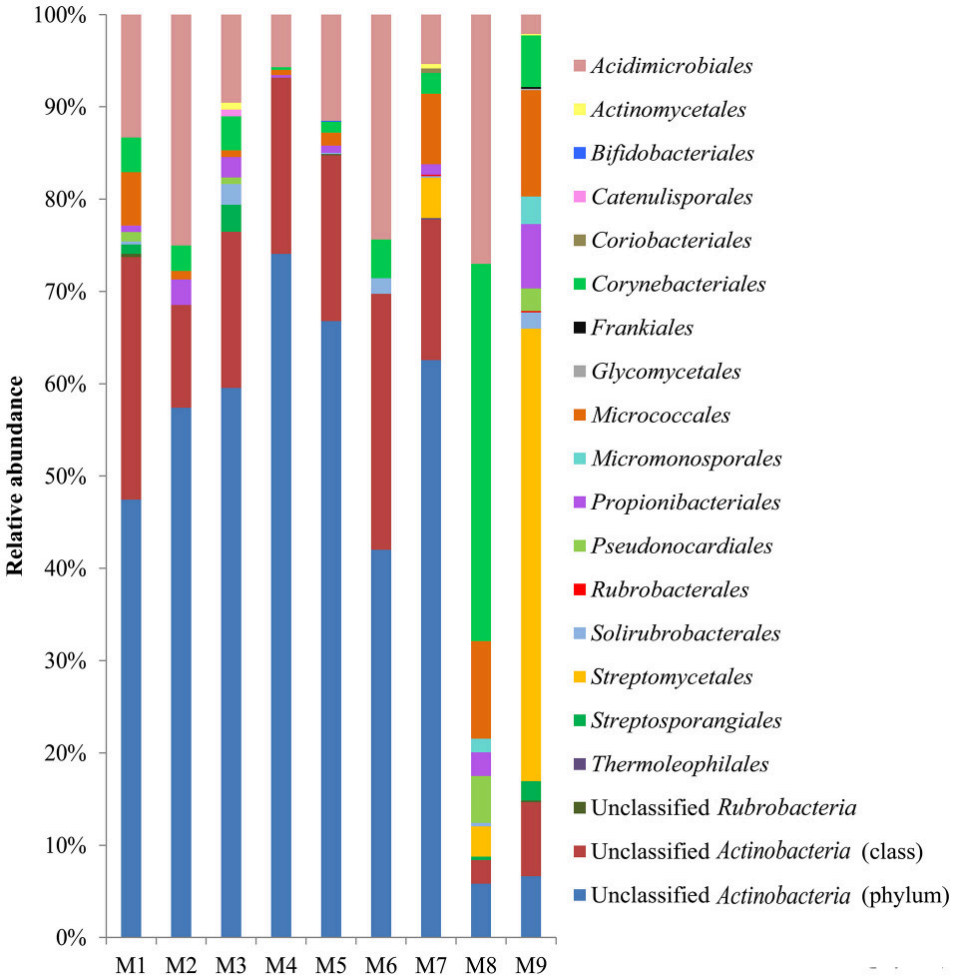
**Actinobacterial community compositions at the order rank in the samples**. The relative abundance represents the proportional frequencies of actinobacterial reads that were classified or unclassified at the order rank, using the RDP classifier, based on the hierarchical classification in the second edition of Bergey's Manual of Systematic Bacteriology (Goodfellow et al., 2012).

At the rank of order, 63.2% (8.4–93.2% among the samples) of the actinobacterial reads could not be classified, however, 17 known actinobacterial orders were detected (Figure 3). *Acidimicrobiales* (2.1–27.0%) and *Corynebacteriales* (0.3–40.9%) were the most widely distributed, present in all samples, followed by *Micrococcales* (0.6–11.5%) and *Propionibacteriales* (0.7–7.0%), present in eight samples except for M6. *Streptomycetales* occurred in samples M7–M9, accounting for almost half (49.0%) of the reads in M9 but only 4.4 and 3.3% in M7 and M8, respectively. In contrast to the non-hydrothermal field sediment samples M1–M7, where unclassified actinobacteria at the order rank accounted for more than 68.5%, the hydrothermal field sediment sample M8 contained *Corynebacteriales* (40.9%) as the most abundant order and had a much higher relative abundance of *Acidimicrobiales, Micrococcales, Micromonosporales, Streptomycetales*, and *Pseudonocardiales*. The hydrothermal field bottom water sample M9 contained the highest number (13) of known orders.

Only 28.5% of the reads could be further classified into the known families (28 in total), and the majority of unclassified reads at the family rank belonged to the order *Acidimicrobiales*. None of the known families were discovered in all samples and seven were present in only one of the samples (Figure S2). The families *Acidimicrobiaceae, Iamiaceae*, and *Propionibacteriaceae* were obtained from eight samples, followed by *Micrococcaceae* and *Nocardiaceae* from seven samples, and *Microbacteriaceae* and *Tsukamurellaceae* from six samples. These families were relatively abundant in the actinobacterial community (>1% of the total actinobacterial reads; Figure S2). All the *Streptomycetales* reads could be further classified into *Streptomycetaceae*, which was also abundant.

**Figure 4:**
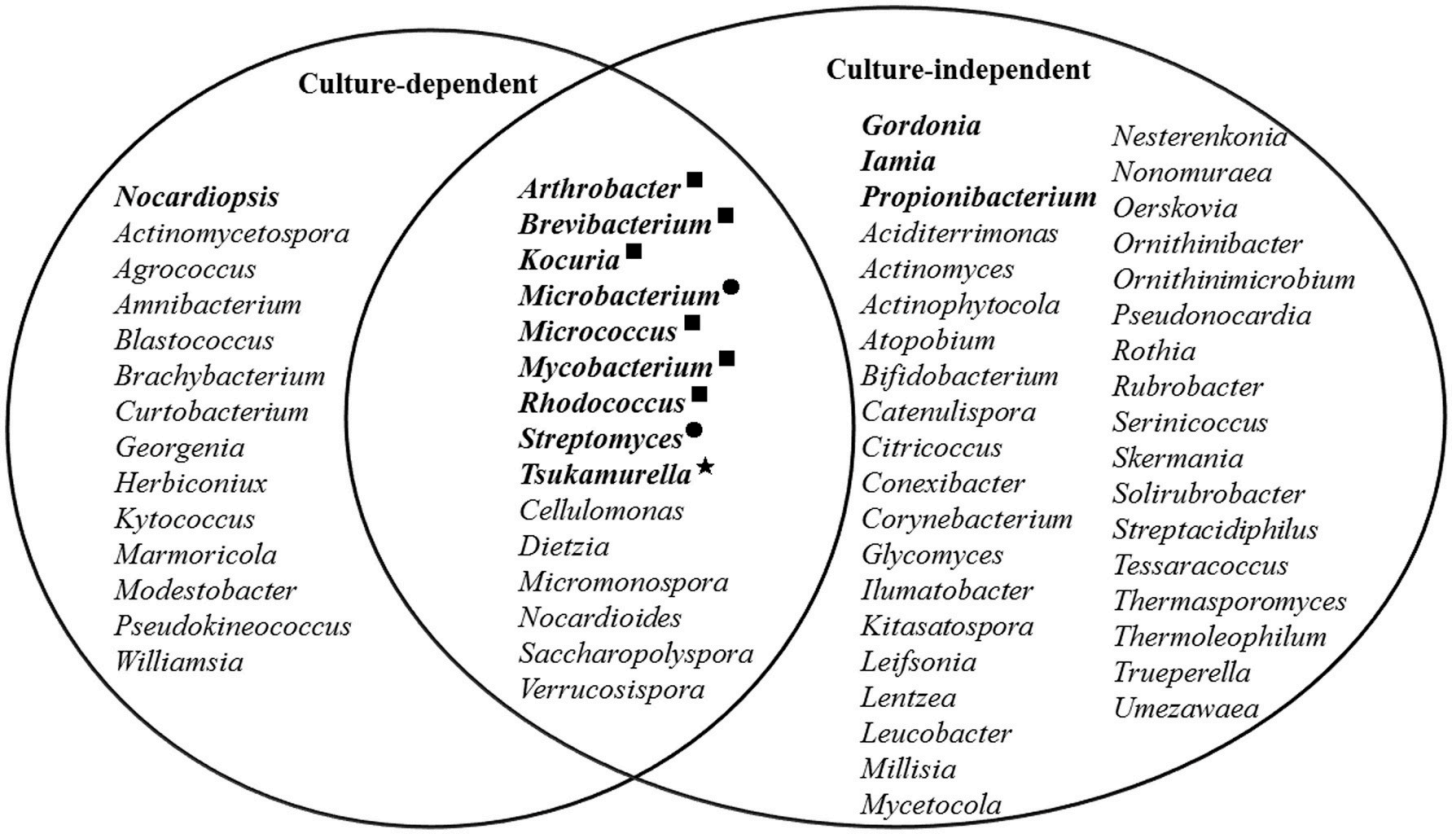
**Actinobacterial genera detected by culture-dependent and -independent methods**. The most abundant genera are shown in bold, and the black dots, star, and squares indicate those that were abundant in both methods, pyrosequencing only, and cultivation only, respectively.

When looking at the genus level, at least 52 known genera were detected. Among them, 35 genera represented non-filamentous actinobacteria according to the previous descriptions of the genera (Goodfellow et al., 2012), accounting for nearly half of the total known actinobacterial reads. Six genera were found to be abundant (Figure 4), of which *Iamia, Microbacterium, Propionibacterium*, and *Tsukamurella* were reported to be non-filamentous, while *Gordonia* and *Streptomyces* were filamentous (Goodfellow et al., 2012).

The actinobacterial sequences unclassified at the family level using the RDP classifier, belonging to 513 OTUs and accounting for 71.5% of actinobacterial reads, were further classified using SINA Alignment Service based on the SILVA database. Nearly half of these OTUs (241 OTUs) were classified as OM1 group of *Acidimicrobiales*, which is also an uncultured group (Yilmaz et al., 2016), accounting for 81.3% of the unclassified reads. The identities to the reference OM1 sequences were 85.5–100.0%. Most of the other OTUs (173 OTUs accounting for 10.3% of the unclassified reads) remained unclassified and showed 70.8–94.6% identities to the unclassified sequences in the SILVA database. Only 99 OTUs could be further classified. Among them, 54 OTUs were classified to known or candidatus genera or families including *Acidothermus, Arthrobacter, Brevibacterium, Candidatus Microthrix, Corynebacterium, Crossiella, Frankiaceae, Geodermatophilus, Jatrophihabitans, Mycobacterium, Pseudonocardiaceae, Rothia, Solirubrobacterales 480-2, Solirubrobacterales Elev-16S-1332, Streptomyces*, and *Sva0996 marine group*; 38 OTUs were classified to known or candidatus orders including *Acidimicrobiales, Frankiales, Gaiellales, Micrococcales, PAUC43f marine benthic group*, and *Solirubrobacterales*; and 7 OTUs were classified to known or candidatus classes of *Actinobacteria, OPB41, TakashiAC-B11*, and *Thermoleophilia*.

### Correlating actinobacterial community compositions with environmental factors

**Figure 5:**
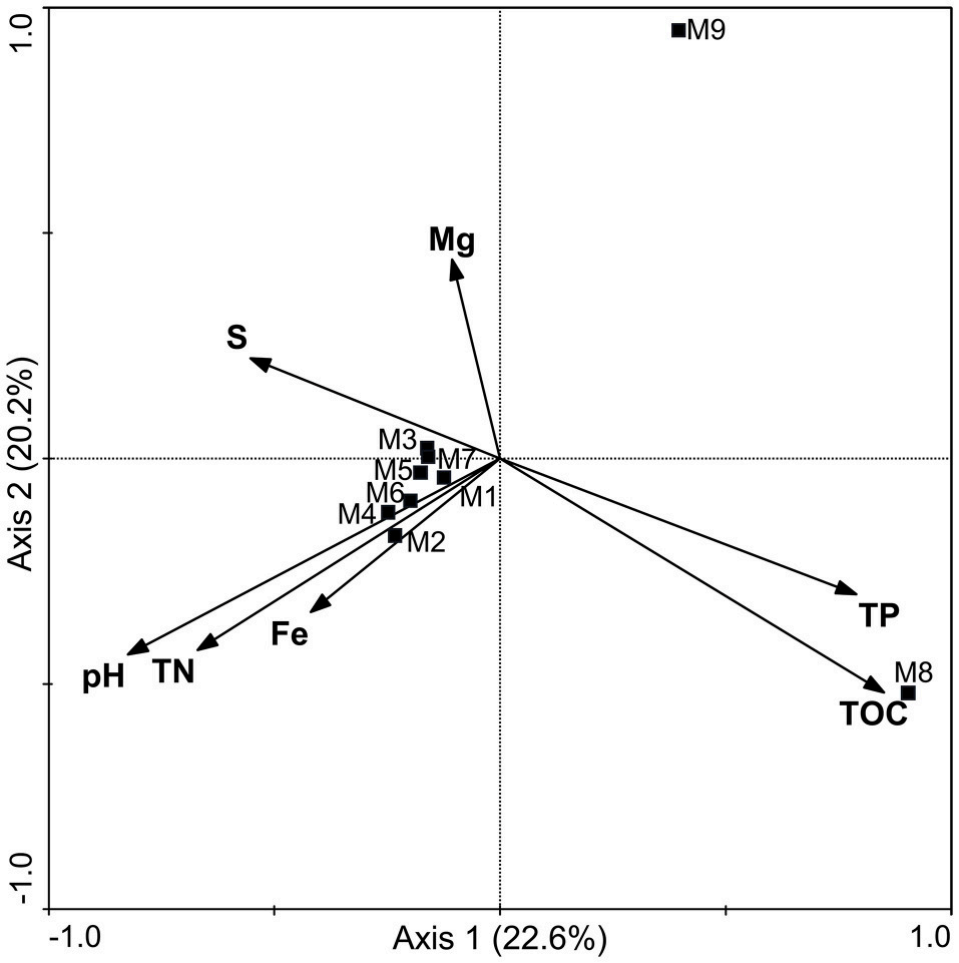
**Canonical correspondence analysis of actinobacterial OTUs (cutoff = 0.03) detected by pyrosequencing**. Arrows indicate the environmental variables and the communities in the samples are represented by squares. The distance between the squares represents the dissimilarity of the communities. The length of the arrows and the included angle of the axes indicate the influence of the specified environmental factors on the community structure. TN, total nitrogen; TP, total phosphorus; TOC, total organic carbon.

The diversity analysis showed that the relative abundance of actinobacterial taxa varied in different samples (Figure 3, Figures S1, S2). PCoA analysis at the 0.03 OTU level further confirmed obvious variations in the actinobacterial community among the samples (Figure S3), a result in line with that of physicochemical factors (Figure 2). Simple Mantel tests between the distance matrix of actinobacterial communities and distance matrices of environmental variables showed that the community structure of the samples was significantly correlated to pH, TOC, TP, Ca and TN (*P* < 0.05; Table 3), where pH showed the highest correlation (*r* = 0.813, *P* = 0.020). When considering only the non-hydrothermal field samples, the actinobacterial community structure was merely correlated with Ca and pH (*P* < 0.05). Canonical correspondence analysis, which was chosen as the appropriate mathematical model in consideration of the axis lengths in Detrended correspondence analysis (Lepš and Šmilauer, 2003), was also performed to identify the major environmental factors shaping the actinobacterial community structure. The result demonstrated a significant model (*P* = 0.042) with seven environmental variables (pH, TOC, Fe, Mg, S, TN, and TP), which explained 91.2% variance of the actinobacterial community compositions with 22.6 and 20.2% explained by the first and second axes, respectively (Figure 5). Among these seven variables, pH and TOC were the most important environmental factors. Geographic distance was not a significant factor because the *P*-values were > 0.05 in both the Mantel test and ordination analysis.

**Table 3:** **Correlations between the actinobacterial community structure (0.03 OTU level) and environmental variables by Mantel tests**.

**Factor**	***r***	***P***
**pH**	**0.813**	**0.020**
**TOC**	**0.640**	**0.027**
**TP**	**0.526**	**0.041**
**Ca**	**0.523**	**0.040**
**TN**	**0.487**	**0.022**
Ln_Distance	−0.167	0.726
EC	0.390	0.170
Fe	0.016	0.286
Mg	−0.005	0.315
Mn	−0.122	0.388
S	0.121	0.291
NO-N	0.140	0.240

*The significant correlations are shown in bold*.

The relative abundance of the phylum *Actinobactreia* showed a significantly negative correlation with Fe (*r* = −0.676, *P* = 0.045). The relationship between the relative abundance of the major groups of actinobacteria and environmental factors was further analyzed. The class *Actinobactreia* showed significant correlations with Mn (*r* = −0.849, *P* = 0.004), pH (*r* = −0.715, *P* = 0.004), NO-N (*r* = 0.754, *P* = 0.019), Fe (*r* = −0.724, *P* = 0.027), and Mg (*r* = 0.676, *P* = 0.046). At the order rank, significant correlations were found between *Corynebacteriales* and TP (*r* = 0.838, *P* = 0.005), *Micrococcales* and Ca (*r* = −0.920, *P* = 0.000), pH (*r* = −0859, *P* = 0.003), TN (*r* = −0.749, *P* = 0.020), Fe (*r* = −0.715, *P* = 0.030), and TP (*r* = 0.713, *P* = 0.031), and between *Propionibacteriales* and electric conductance (*r* = 0.904, *P* = 0.001), TN (*r* = −0.862, *P* = 0.003), and pH (*r* = −0.817, *P* = 0.007). For the known actinobacterial families, only *Micrococcaceae* and *Propionibacteriaceae* showed negative correlations to TN and pH and a positive correlation to electric conductance (Figure S4). The results also indicated that unclassified *Actinobacteria* (phylum) positively correlated with pH and TN, unclassified *Actinobacteria* (class) correlated with TN, NO-N, and pH, and unclassified *Acidimicrobiales* highly correlated with Fe (Figure S4). Some of the OTUs within these unclassified/uncultured groups were more influenced than others, including the OTUs detected in most of the samples. For instance, OTU00513 and OTU00620 showed significant correlations with pH (*r* = 0.782, *P* = 0.013; *r* = 0.692, *P* = 0.039) and TN (*r* = 0.689, *P* = 0.040; *r* = 0.764, *P* = 0.017), OTU00305 and OTU00066 significantly correlated to TN (*r* = 0.791, *P* = 0.011; *r* = 0.712, *P* = 0.032), OTU00085 and OTU00624 correlated to NO-N (*r* = 0.771, *P* = 0.015; *r* = 0.721, *P* = 0.028), and OTU00183 had a highly significant correlation with Fe (*r* = 0.854, *P* = 0.003). All the above mentioned OTUs were classified to *Acidimicrobiales* OM1 clade using the SILVA database.

Besides the geochemical parameters, correlations between the relative abundance of the phylum *Actinobactreia* and other bacterial and archaeal phyla present in the samples were also evaluated. The result showed a significant correlation between *Actinobactreia* and *Chloroflexi* (*r* = 0.678, *P* = 0.045).

### Diversity of culturable actinobacteria

**Figure 6:**
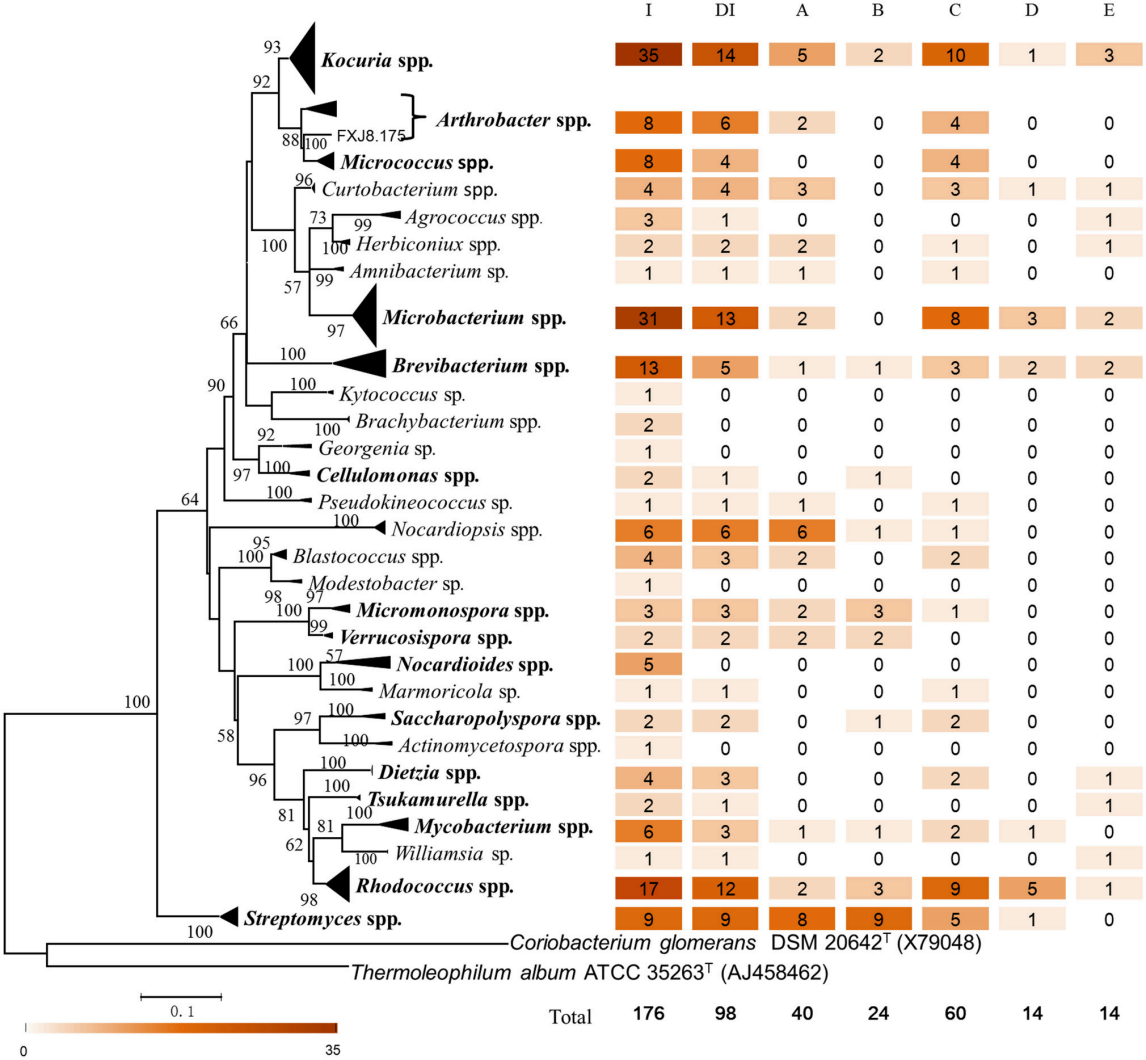
**Phylogenetic diversity, taxonomic affiliation and degradation capacity of culturable actinobacterial isolates**. The maximum-likelihood tree was based on nearly full-length 16S rRNA gene sequences of the isolates and closely related type strains. The GTR+G+I evolutionary model was selected to construct the tree. Coriobacterium glomerans DSM 20642T and Thermoleophilum album ATCC 35263T were set as outgroups. Numbers at branch nodes are percentages of bootstrap replicates of 1000 resamplings (only values above 50% are shown). The bar represents 0.1 substitutions per nucleotide position. Genera that were also recovered in the pyrosequencing data set are in bold. Numbers following each genus indicate the number of isolates or degrading isolates. I, number of isolates; DI, number of degrading isolates; A, number of cellulose-degrading isolates; B, number of chitin-degrading isolates; C, number of pectin-degrading isolates; D, number of fluoranthene-degrading isolates; E, number of phenanthrene-degrading isolates.

To further investigate the diversity and potential functions of actinobacteria at the SWIR, the samples were subjected to isolation of actinobacterial strains using three pretreatments and eight selective media. A total of 176 actinobacterial strains were purified from the samples. Most of the strains were isolated from ISP 3 (62 strains) and AMM (46 strains) media. The 16S rRNA gene sequences of the isolates were affiliated with 29 genera, 18 families, and 9 orders within the *Actinobacteria* class, of which 15 genera, 13 families and 8 orders were also found in the pyrosequencing data set (Figures 4, 6). The isolates showed 97.3–100% 16S rRNA gene sequence similarities to the closest known actinobacterial species, with the most divergent isolate belonging to *Nocardioides*.

**Figure 7:**
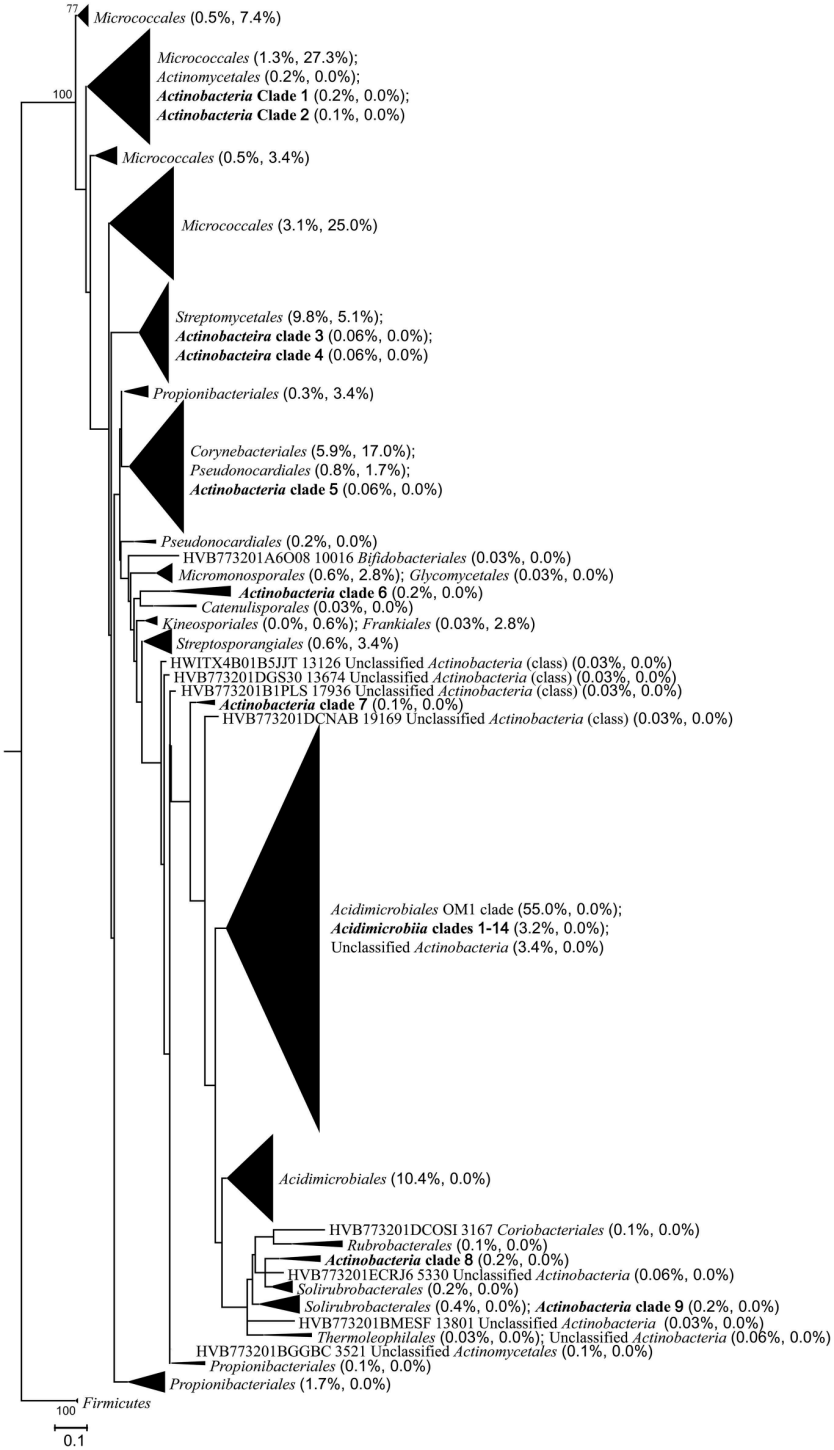
**Maximum-likelihood phylogenetic tree based on partial 16S rRNA gene sequences illustrating the relationships among all the isolates and representative actinobacterial sequences from the pyrosequencing data set**. The tree was constructed using the GRT+G+I model. Firmicutes was set as the outgroup. Taxonomic assignments of the sequences were based on both the RDP and SILVA databases. The tree is displayed at order level. Numbers at branch nodes are percentages of bootstrap replicates of 100 resamplings (only values above 50% are shown). Numbers in the parentheses following each order or clade indicate the relative abundance of pyrosequencing reads and isolates, respectively. The bar represents 0.1 substitutions per nucleotide position.

A total of 77.3% (136/176) of the isolates showed ≥97% 16S rRNA sequence similarities to the pyrosequencing reads, whereas only 10.3% (335/3245) of the *in situ* actinobacterial community were represented by the isolates when mapping the actinobacterial reads against the sequences of the isolates at the 97% similarity cutoff. The phylogenetic analysis on all the isolates and representative actinobacterial sequences from the pyrosequencing data set also supported the above results (Figure 7). Most of the isolates clustered closely with the environmental sequences, formed 8 of the 18 known orders detected in total. Nine known orders contained sequences from only the pyrosequencing but not the isolates, while the order *Kineosporiales* contained only the isolate sequences. On the other hand, most of the unclassified/uncultured pyrosequencing sequences formed independent clades that were distinguished from the isolates, notably the *Acidimicrobiales* OM1 clade which was also distinct from the clade of the order *Acidimicrobiales* and should probably be named as a new order. The phylogenetic tree also showed that there were 14 *Acidimicrobiia* clades and a number of unclassified actinobacterial sequences clustered with the OM1 clade, and there were at least three distinguished *Actinobacteria* clades (*Actinobacteria* clades 6–8) that might respectively represent new orders within the phylum *Actinobacteria* (Figure 7, Figures S5A–S5F).

Among the 15 genera that were present in both the pyrosequencing and cultural data sets, only three (*Streptomyces, Tsukamurella*, and *Microbacterium*) were abundant in pyrosequencing (8.8, 2.1, and 1.5%, respectively); and most of the cultured genera represented rare groups or were absent in pyrosequencing. None of the genera were recovered from all samples. The non-filamentous genera *Kocuria* (35 isolates) and *Microbacterium* (31 isolates), both belonging to the order *Micrococcales*, were the most abundant of the culturable genera. *Microbacterium* isolates were obtained from all samples except M1, while *Kocuria* isolates were from M1, M4, M6, and M9. The highest diversity of isolates was observed for the hydrothermal field bottom water sample M9 (13 genera), followed by sample M4 (12 genera); and the lowest diversity was observed for samples M3 and M7, each affiliated with only four genera (Table 4).

**Table 4:** **Numbers of isolates (and degrading isolates) at the genus level from different samples**.

**Orders**	**Family**	**Genus**	**M1**	**M2**	**M3**	**M4**	**M5**	**M6**	**M7**	**M8**	**M9**	**Total**
*Corynebacteriales*	*Dietziaceae*	*Dietzia*	0	0	1 (1)	0	0	0	1 (0)	0	2 (2)	4 (3)
	*Mycobacteriaceae*	*Mycobacterium*	0	0	0	0	3 (2)	1 (0)	0	1 (1)	1 (0)	6 (3)
	*Nocardiaceae*	*Rhodococcus*	0	1 (1)	0	8 (6)	0	0	5 (2)	1 (1)	2 (2)	17 (12)
	*Tsukamurellaceae*	*Tsukamurella*	0	0	0	0	0	0	0	1 (1)	1 (0)	2 (1)
	*Nocardiaceae*	*Williamsia*	0	0	0	0	1 (1)	0	0	0	0	1 (1)
*Frankiales*	*Geodermatophilaceae*	*Blastococcus*	0	0	0	2 (1)	1 (1)	1 (1)	0	0	0	4 (3)
		*Modestobacter*	0	0	0	0	1 (0)	0	0	0	0	1 (0)
*Kineosporiales*	*Kineosporiaceae*	*Pseudokineococcus*	0	0	0	1 (1)	0	0	0	0	0	1 (1)
*Micrococcales*	*Brevibacteriaceae*	*Brevibacterium*	2 (2)	1 (1)	0	0	0	0	0	4 (2)	6 (0)	13 (5)
	*Bogoriellaceae*	*Georgenia*	0	1 (0)	0	0	0	0	0	0	0	1 (0)
	*Cellulomonadaceae*	*Cellulomonas*	0	0	0	0	1 (0)	0	0	0	1 (1)	2 (1)
	*Microbacteriaceae*	*Agrococcus*	0	0	0	1 (1)	0	0	2 (0)	0	0	3 (1)
		*Amnibacterium*	0	0	0	1 (1)	0	0	0	0	0	1 (1)
		*Curtobacterium*	1 (1)	1 (1)	0	1 (1)	1 (1)	0	0	0	0	4 (4)
		*Herbiconiux*	0	1 (1)	0	0	1 (1)	0	0	0	0	2 (2)
		*Microbacterium*	0	6 (1)	1 (0)	3 (2)	10 (6)	6 (1)	1 (0)	1 (1)	3 (2)	31 (13)
	*Micrococcaceae*	*Arthrobacter*	1 (1)	1 (1)	0	2 (2)	4 (2)	0	0	0	0	8 (6)
		*Micrococcus*	3 (1)	1 (1)	0	1 (0)	0	0	0	0	3 (2)	8 (4)
		*Kocuria*	26 (9)	0	0	3 (1)	0	3 (3)	0	0	3 (1)	35 (14)
	*Dermabacteraceae*	*Brachybacterium*	0	2 (0)	0	0	0	0	0	0	0	2 (0)
	*Dermacoccaceae*	*Kytococcus*	0	0	0	0	1 (0)	0	0	0	0	1 (0)
*Micromonosporales*	*Micromonosporaceae*	*Micromonospora*	2 (2)	0	0	1 (1)	0	0	0	0	0	3 (3)
		*Verrucosispora*	0	0	0	0	0	0	0	0	2 (2)	2 (2)
*Propionibacteriales*	*Nocardioidaceae*	*Marmoricola*	0	1 (1)	0	0	0	0	0	0	0	1 (1)
		*Nocardioides*	0	0	0	1 (0)	4 (0)	0	0	0	0	5 (0)
*Streptosporangiales*	*Nocardiopsaceae*	*Nocardiopsis*	0	0	0	0	0	1 (1)	0	2 (2)	3 (3)	6 (6)
*Pseudonocardiales*	*Pseudonocardiaceae*	*Actinomycetospora*	0	1 (0)	0	0	0	0	0	0	0	1 (0)
		*Saccharopolyspora*	0	0	1 (1)	0	0	0	0	0	1 (1)	2 (2)
*Streptomycetales*	*Streptomycetaceae*	*Streptomyces*	4 (4)	0	2 (2)	0	0	0	0	1 (1)	2 (2)	9 (9)
No. of isolates (degrading isolates)			39 (20)	17 (8)	5 (4)	25 (17)	28 (14)	12 (6)	9 (2)	11 (9)	30 (18)	176 (98)
No. of genera (degrading genera)			7 (7)	11 (8)	4 (3)	12 (10)	11 (7)	5 (4)	4 (1)	7 (7)	13 (10)	29 (23)

### Biodegradation activity of actinobacterial isolates

The majority of isolates (98 of 176) demonstrated activity in the degradation of refractory organics, i.e., cellulose (40 isolates, 22.7%), chitin (24 isolates, 13.6%), pectin (60 isolates, 34.1%), fluoranthene (14 isolates, 8.0%), and phenanthrene (14 isolates, 8.0%; Figure 6). The degrading isolates spread across 23 genera, with the pectin-degrading strains the most diverse (18 genera), followed by the cellulose degraders (15 genera), chitin degraders (10 genera), phenanthrene degraders (10 genera), and fluoranthene degraders (7 genera; Figure 6). Among them, 41 isolates belonging to 15 genera showed activity toward more than one organic substance. Nearly half of the degrading isolates were distributed in four genera, which were *Kocuria* (14 strains), *Microbacterium* (13 strains), *Rhodococcus* (12 strains), and *Streptomyces* (9 strains); and isolates classified within each of the genera *Brevibacterium, Kocuria*, and *Rhodococcus* showed degradation activity toward all of the organics tested. Almost all of the *Streptomyces* isolates showed activities toward cellulose (8 of 9) and chitin (9 of 9). Pectin degraders were mainly from the genera *Kocuria, Microbacterium, Rhodococcus*, and *Streptomyces*, of which *Rhodococcus* also accounted for >1/3 of the fluoranthene degraders (Figure 6). Cellulolytic and pectinolytic strains could be found in all nine samples. Furthermore, 9 of the 11 isolates and all 7 genera from M8 could degrade the organics, showing the highest activity rate among the samples (Table 4).

## Discussion

The results showed a high diversity and a relatively comprehensive community structure of actinobacteria in the deep-sea surface sediments and water along the SWIR, which significantly correlated with environmental factors. The biodegradation activity toward refractory organics demonstrated by most of the diverse actinobacterial isolates suggests their potential ecological functions and biotechnological uses.

### Actinobacteria in the deep sea along the SWIR are highly diverse

The 16S rRNA gene pyrosequencing data showed that the relative abundance of actinobacteria in the sediments collected from the SWIR was higher than that found in the sediments of the deep Arctic and Atlantic Oceans (0.1–3.0%, 0–2.32 mbsf), but less than that reported in the deep Pacific Ocean (about 10%, 0–350 mbsf) (Inagaki et al., 2006; Schauer et al., 2010; Jorgensen et al., 2012). Our results also indicate that many unclassified actinobacterial taxa may exist in the deep sea along the SWIR, particularly in the surface sediments in non-hydrothermal fields (Figures 3, 7, Figures S1, S2). While >80% of the actinobacterial unclassified reads were assigned to *Acidimicrobiales* OM1 clade using the SILVA database, most of these sequences, according to the recent data of Yilmaz et al. (2016), should belong to the uncultured *Actinobacteria*.Order3 which is distinct from the OM1 clade (*Actinobacteria*.Order2 in Yilmaz et al., 2016). Most of the remaining sequences were assigned into the classes *Actinobacteria*, similar to that reported for the surface sediments of the deep Arctic Ocean (Zhang et al., 2014). Members of the two classes have been reported to be widely distributed in various habitats; however, only members of *Actinobacteria* have been frequently isolated from the marine environment (Maldonado et al., 2005; Duncan et al., 2014; Zhang et al., 2014), which is also the case in our study. Most members of the class *Acidimicrobiia* are uncultured, and *Acidimicrobiaceae*, and *Iamiaceae* are the only two described families in this class (Jensen and Lauro, 2008; Goodfellow et al., 2012). Therefore, it is not strange to find in our study that a large number of the sequences of *Acidimicrobiia* could not be classified into a known family and *Iamiaceae*, a neutrophilic family, presented in eight samples including both the sediments and the bottom water. Interestingly, *Acidimicrobiaceae*, members of which prefer to grow at around pH 2 and have been isolated only from geothermal sites and acidic mine waters (Goodfellow et al., 2012), also occurred in all the SWIR sediment samples with non-negligible percentages (0.7−4.6%, Figure S2), although the pH of the samples was 7.77−9.11 (Table 1).

Compared to the results of previous studies on marine actinobacterial diversity, which reported only 4−12 genera or 14 families, much more genera and families were detected in the actinobacterial community at the SWIR using either pyrosequencing or cultivation method (Figure 4, Table S2), with at least 66 known genera and 33 families detected in total (Figure 4). Most of the marine actinobacterial genera and families detected in previous studies were recovered in our results (Figure 4, Table S2), but the most abundant genera detected in deep Arctic marine surface sediments were largely different from those identified here (Table S2). Moreover, most of the known genera (46 of 66) detected at the SWIR were likely to be non-filamentous based on the previous descriptions (Goodfellow et al., 2012), similar to the finding in deep Arctic marine surface sediments (Zhang et al., 2014). In agreement with this result, most of our isolates (74.4%) were non-filamentous actinobacteria, an observation in contrast to previous reports on shallow sediments, where filamentous actinobacteria, especially *Micromonospora, Pseudonocardia*, and *Streptomyces*, were most frequently cultivated (Maldonado et al., 2005; Bredholdt et al., 2007; Zhang et al., 2014). This difference might primarily result from the change in hydrostatic pressure, which is an important parameter in shaping the microbial community structure in the ocean and leads to small cocci being the most abundant morphotype of high-pressure-surviving microbes (Marietou and Bartlett, 2014). In addition, 53 isolates showed < 99% 16S rRNA gene similarity to their closest type strains and therefore likely represented novel species according to the recommended cutoff value for actinobacterial species delineation (Stach et al., 2003a).

### The actinobacterial community structure at the SWIR significantly correlates to environmental factors

Researchers have gradually formed a consensus on the biogeography of marine actinobacteria (Ward and Bora, 2006). However, the related factors that contribute to shape the actinobacterial community structure, especially in the deep ocean, are still not clear. This study showed that pH and Ca were significant factors along the SWIR, whether the two hydrothermal field samples, M8 and M9, were considered or not. It has been demonstrated that pH was an important contributor to actinobacterial/prokaryotic community variations in soil (pH 3.5–8.8), salt lakes (pH 7.5–10.5), and hot springs (pH 5.0–10.0; Lauber et al., 2009; Pagaling et al., 2009; Valverde et al., 2012). In our study, a less-massive change in pH from 7.47 to 9.11 demonstrated a significantly high influence on the actinobacterial community structure (Table 3), indicating that the deep-sea actinobacterial composition is sensitive to pH. This result was further supported by the evidence that pH significantly affected the relative abundance of the class *Actinobactreia*, the orders *Corynebacteriales, Micrococcales*, and *Propionibacteriales*, the families *Micrococcaceae* and *Propionibacteriaceae*, and unclassified *Actinobacteria* (both class and phylum) at the SWIR. It was not unexpected that Ca, which was an essential nutrient and involved in pH buffering, was an environmental determinant of the community structure as well. Our study also showed for the first time that TOC, TP, and TN, which represented nutrients, were significantly correlated to actinobacterial community structure in the deep sea. Dissolved organic carbon and TP were previously reported to be related to the freshwater actinobacterial community structure in the brackish northern Baltic Sea (Holmfeldt et al., 2009). Total carbon, TN, and P were also reported to be significantly correlated to the relative abundance of actinobacteria in soils (Liu et al., 2014; Zhao et al., 2014). Taken together, pH and the above mentioned nutrients are likely to be the common factors that shape actinobacterial community structures in different habitats. Interestingly, some environmental variables significantly influenced the relative abundance of a few unclassified actinobacterial groups (Figure S4). This finding suggests that the responses of these unknown taxa, particularly the specific OTUs that were more influenced, to local environments may relate to their critical requirements for pH and nutrients.

The observation that the actinobacterial community structure was not affected by the geographic distance might be attributed to the fact that many actinobacteria can produce stress-resistant spores and possess various metabolic capabilities, characteristics that promoted their long-distance dispersal and survival in the resultant conditions encountered. Nevertheless, this observation may be also due to that our samples were not collected at a large spatial scale (< 1000 km in geographic distance), because the scale of sampling was suggested to affect the relative contributions of spatial distance and environmental factors to microbial distribution (Martiny et al., 2006, 2011). Hence, extensive sampling at a larger geographic scale with more hydrothermal fields is still needed to determine whether actinobacterial distribution along the Indian Ridge and even the global deep ocean is controlled by historical or contemporary environmental factors or both.

### Actinobacteria from the SWIR show potential ecological functions and biotechnological applications

Although, the concentrations of PAHs and polysaccharides of the samples were not determined in this study due to the difficulty of sampling and the limited amount of samples we obtained, these organics have been reported to be common in the deep ocean (Simoneit and Fetzer, 1996; Dong et al., 2015). More than half of the actinobacterial isolates obtained from this research showed the capacity to degrade organic matter, suggesting that actinobacteria may contribute to the substance and energy flows in biogeochemical cycles in the deep ocean. We found a high diversity of the marine actinobacterial degraders, with 20 polysaccharide-degrading genera and 12 PAH-degrading genera (Figure 6). Most of these genera, with the exception of *Streptomyces, Nocardiopsis*, and *Dietzia*, have not been reported to contain members inhabiting deep-sea environments whilst possessing organic degradation capacity. The genera that could degrade different refractory organics may have a better chance to thrive in a range of deep-sea habitats, as exemplified by *Kocuria, Microbacterium, Mycobacterium, Rhodococcus*, and *Streptomyces*, which spread in most of the samples as indicated by the combined results of culture-dependent and -independent analyses. Hydrolytic enzyme profiles have been analyzed for marine actinobacteria from shallow sediments, and the results indicated that *Streptomyces* had a better ability than the other genera to degrade cellulose, chitin, and pectin (Veiga et al., 1983; Augustine et al., 2013). Among the 23 degrading genera we obtained, *Streptomyces* was again the most important contributor to the degradation of chitin and cellulose. The result that all of the *Streptomyces* isolates could degrade chitin is consistent with the report that a *Streptomyces* strain has at least one and usually multiple chitinase genes and genes of the chitin-binding proteins are also widely distributed in *Streptomyces* (Williamson et al., 2000). The observation that seven and ten actinobacterial genera in this study could degrade fluoranthene and phenanthrene, respectively, suggested a potential for discovering highly-efficient PAH degraders, which may fuel further research into the biodiversity of PAH-degrading actinobacteria in the deep marine environments.

The high abundance of the order *Corynebacteriales* in the hydrothermal field sediment M8 may be explained by the fact that many members of *Corynebacteriales* are degraders of xenobiotica (e.g., PAHs) and excellent survivors of unfavorable conditions (Bock et al., 1996; Willumsen et al., 2001). PAHs and heptaheptacontane are common in hydrothermal sediments (Simoneit and Fetzer, 1996), and the genera *Gordonia, Mycobacterium, Rhodococcus*, and *Tsukamurella* within *Corynebacteriales* have been reported to be highly efficient hydrocarbon degraders (Peczynska-Czoch and Mordarski, 1988; Daane et al., 2001; García-Díaz et al., 2013). This is also supported by the cultivation results of our study, where all five genera (*Dietzia, Mycobacterium, Rhodococcus, Tsukamurella*, and *Williamsia*) of *Corynebacteriales* demonstrated PAH degradation activity (Figure 6).

As most of the secondary metabolite-producing actinobacteria were filamentous (so-called actinomycetes; Tiwari and Gupta, 2012), while most of our actinobacterial isolates were non-filamentous, we did not focus on bioactive compounds of the isolates in this study. Actually we had conducted a primary screening of antimicrobial activity on 37 isolates of nine genera and found that only two isolates belonged to *Micromonospora* and *Streptomyces*, respectively, showed inhibitory activity against *Micrococcus luteus* only. Given the high biodegradation activity of the diverse isolates, it is likely that the ability to utilize refractory organics is important for the survival of actinobacteria in the deep sea. Recent PICRUSt predictions for the uncultured actinobacterial orders in deep pelagic zones also suggested their potential for refractory organic degradation in terms of carbon metabolism (Yilmaz et al., 2016). This characteristic implied the potential roles of actinobacteria in the recycling of organic matter in the deep-sea environments, which might enhance their competitive ability *in situ*.

## Author contributions

YH, PC, XD, and LH designed the study. LS collected the samples. PC, LZ, XG, LX, and YZ conducted the lab work. PC, YH, LL, YW, and JW analyzed the data. YH, PC, XD, and LH wrote the paper.

### Conflict of interest statement

The authors declare that the research was conducted in the absence of any commercial or financial relationships that could be construed as a potential conflict of interest.

## Supplementary material

Figure S1 **Actinobacterial community compositions at the class rank in the samples**. The relative abundance represents the proportional frequencies of actinobacterial reads that were classified or unclassified at the class rank, using the RDP classifier, based on the hierarchical classification in the second edition of *Bergey's Manual of Systematic Bacteriology* (Goodfellow et al., 2012).

Figure S2 **Actinobacterial community compositions at the family rank in the samples**. The relative abundance represents the proportional frequencies of actinobacterial reads that were classified or unclassified at the family rank, using the RDP classifier, based on the hierarchical classification in the second edition of *Bergey's Manual of Systematic Bacteriology* (Goodfellow et al., 2012). The most abundant families are shown in bold.

Figure S3 **PCoA representing the similarity of the actinobacterial community compositions in the samples detected by pyrosequencing at the 0.03 OTU level**.

Figure S4 **The relationships between the relative abundance of some of the major groups of actinobacteria and environmental factors**. EC, electric conductance; TN, total nitrogen; NO-N, nitrate nitrogen.

Figure S5 **Parts of the zoom-in phylogenetic tree of Figure 7 showing the relationships between the 14 *Acidimicrobiia* clades and the OM1 clade**. Taxonomic assignments of the sequences were based on both the RDP and SILVA databases. Numbers at branch nodes are percentages of bootstrap replicates of 100 resamplings (only values above 50% are shown). Numbers in the parentheses following each clade indicate the relative abundance of pyrosequencing reads and isolates, respectively. Bar = nucleotide changes per site.

Table S1 **The geographical distance matrix**.

Table S2 **Summary of the analyses on actinobacterial diversity in shallow and deep seas using culture-dependent and -independent methods**.

## Abbreviations

NO-N: Nitrate Nitrogen
S: Sulphur
SWIR: Southwest Indian Ridge
TN: Total Nitrogen
TOC: Total Organic Carbon
TP: Total Phosphorus.
