# Supplementary figures and images for: Diversity, Biogeography, and Biodegradation Potential of Actinobacteria in the Deep-Sea Sediments along the Southwest Indian Ridge
Source: Front Microbiol. 2016 Aug 29;7:1340. doi: 10.3389/fmicb.2016.01340 (PMC5002886; doi:10.3389/fmicb.2016.01340)

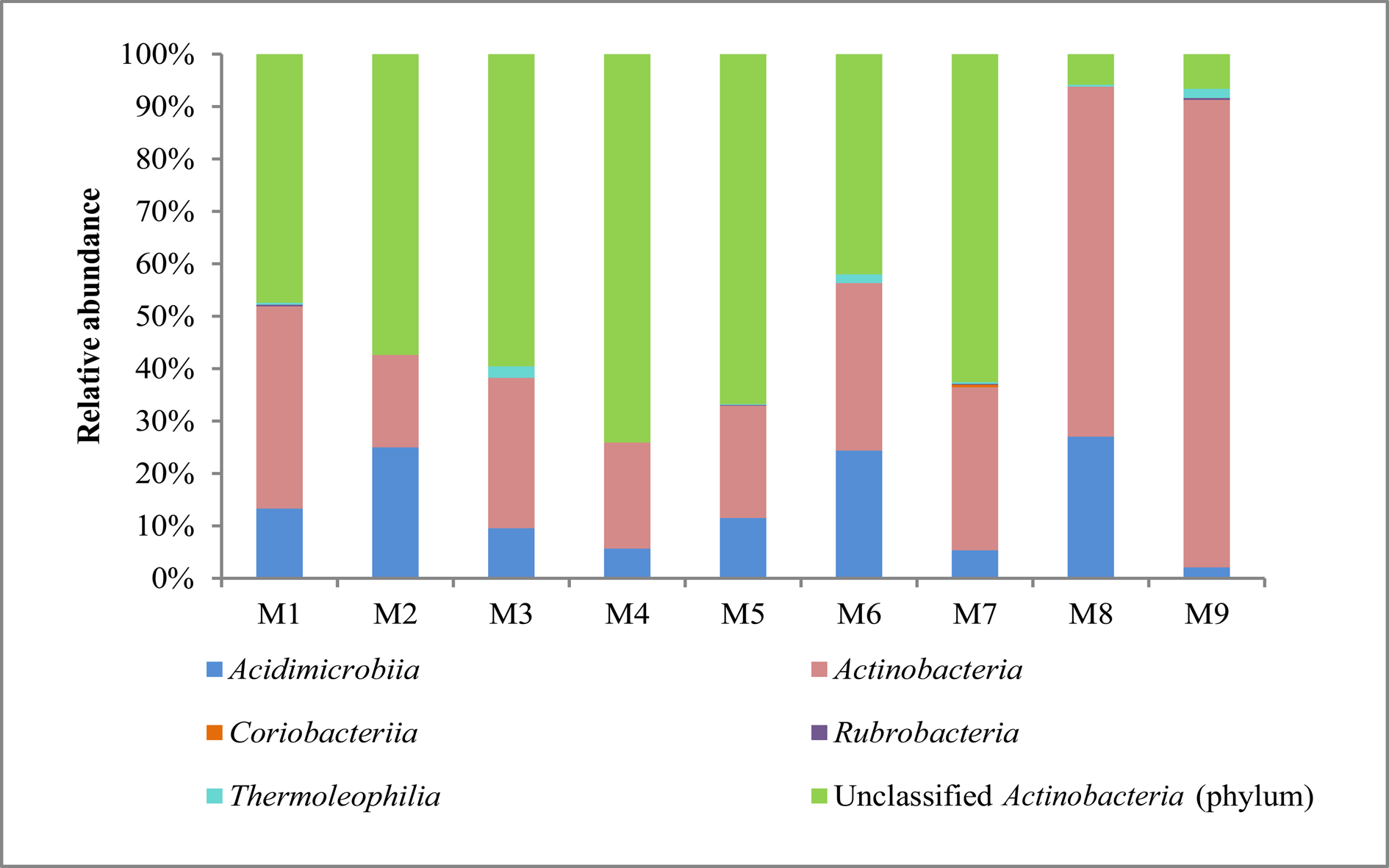

Supplement: Figure S1 — Actinobacterial community compositions at the class rank in the samples. The relative abundance represents the proportional frequencies of actinobacterial reads that were classified or unclassified at the class rank, using the RDP classifier, based on the hierarchical classification in the second edition of Bergey's Manual of Systematic Bacteriology (Goodfellow et al., 2012). [file Image1.TIF]

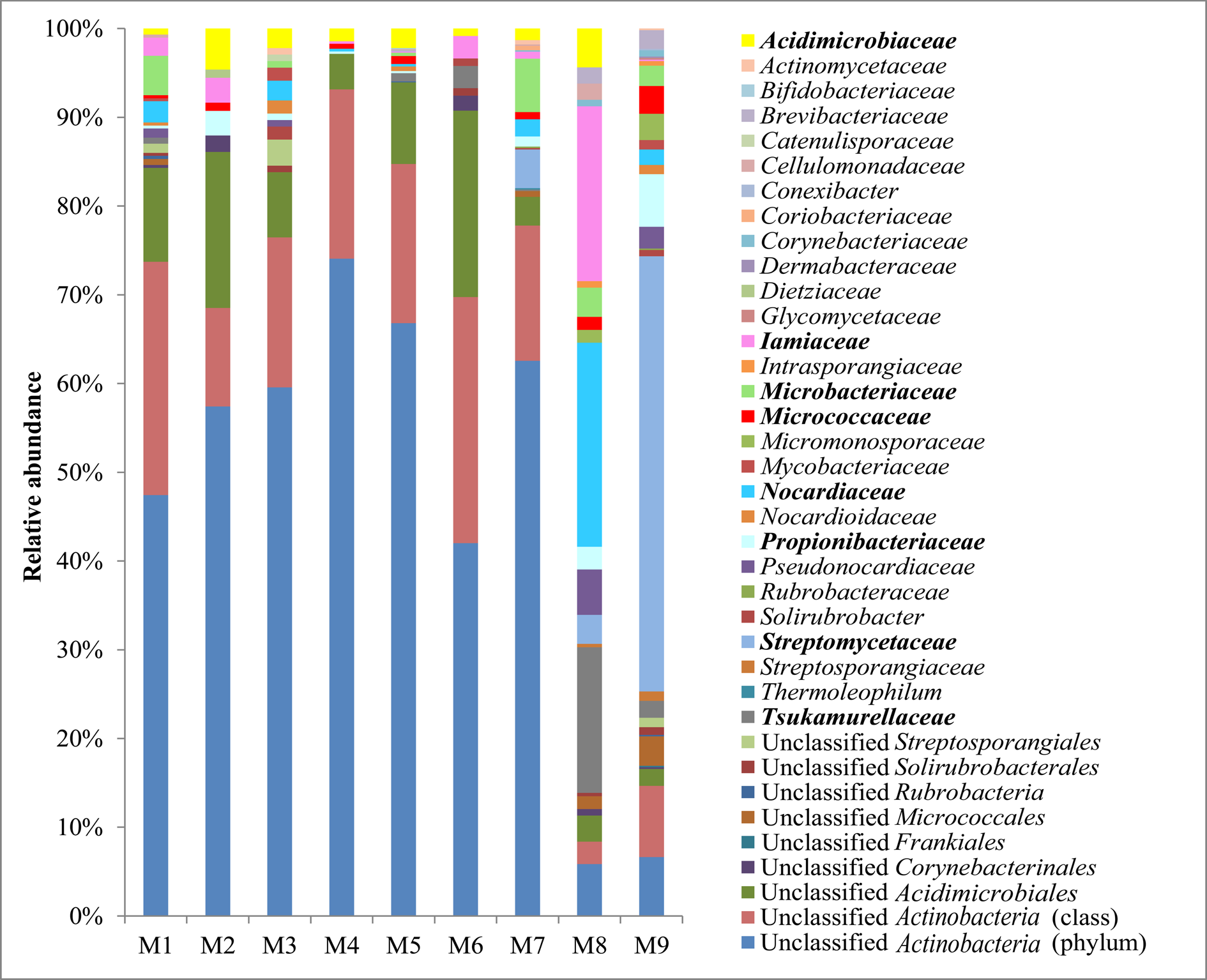

Supplement: Figure S2 — Actinobacterial community compositions at the family rank in the samples. The relative abundance represents the proportional frequencies of actinobacterial reads that were classified or unclassified at the family rank, using the RDP classifier, based on the hierarchical classification in the second edition of Bergey's Manual of Systematic Bacteriology (Goodfellow et al., 2012). The most abundant families are shown in bold. [file Image2.TIF]

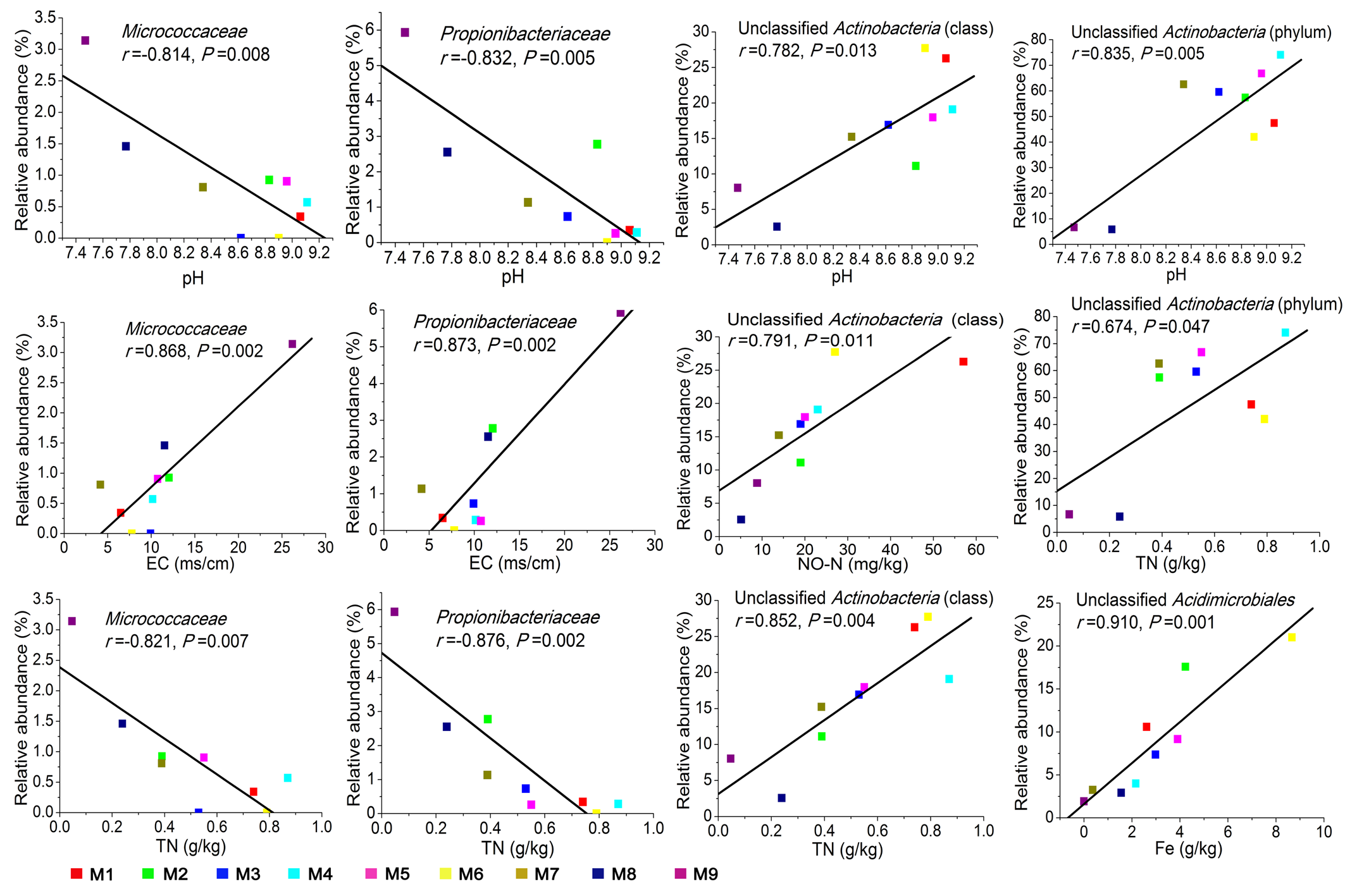

Supplement: Figure S4 — The relationships between the relative abundance of some of the major groups of actinobacteria and environmental factors. EC, electric conductance; TN, total nitrogen; NO-N, nitrate nitrogen. [file Image4.TIF]

**A**

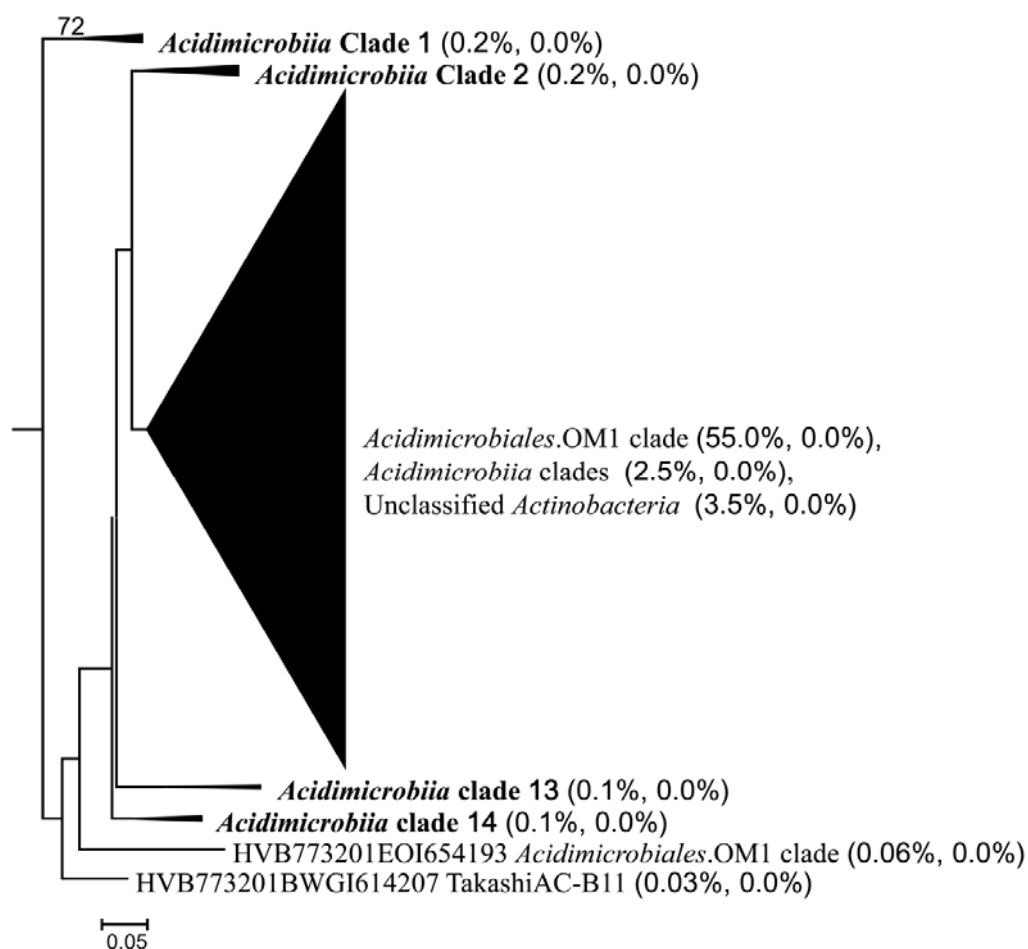

**B**

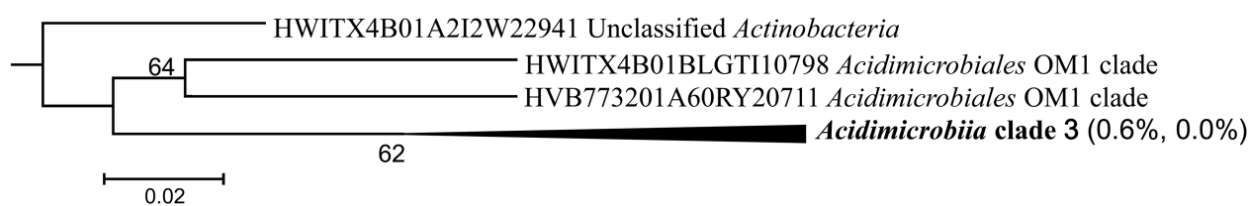

**C**

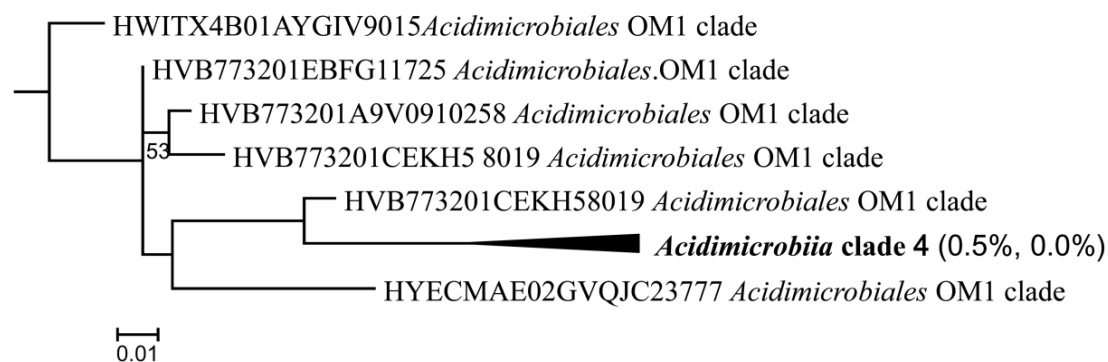

**D**

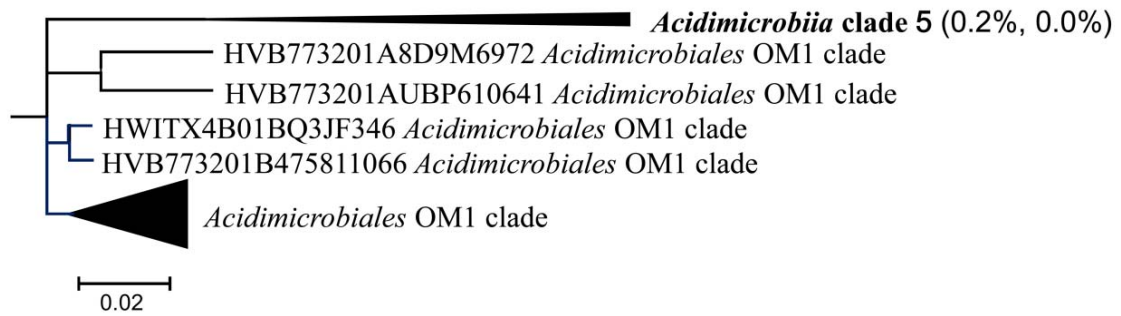

**E**

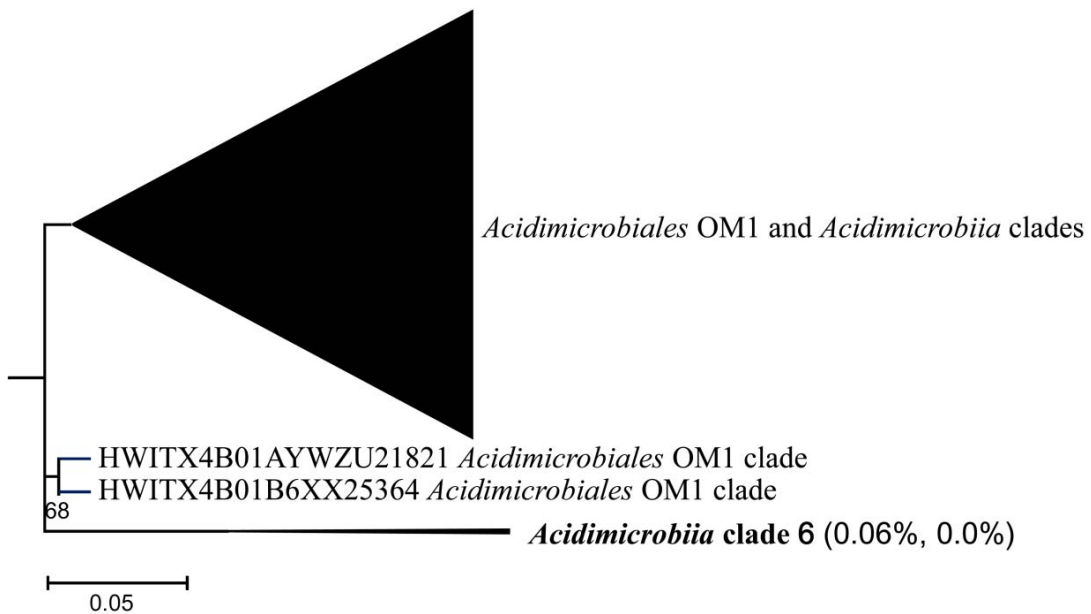

**F**

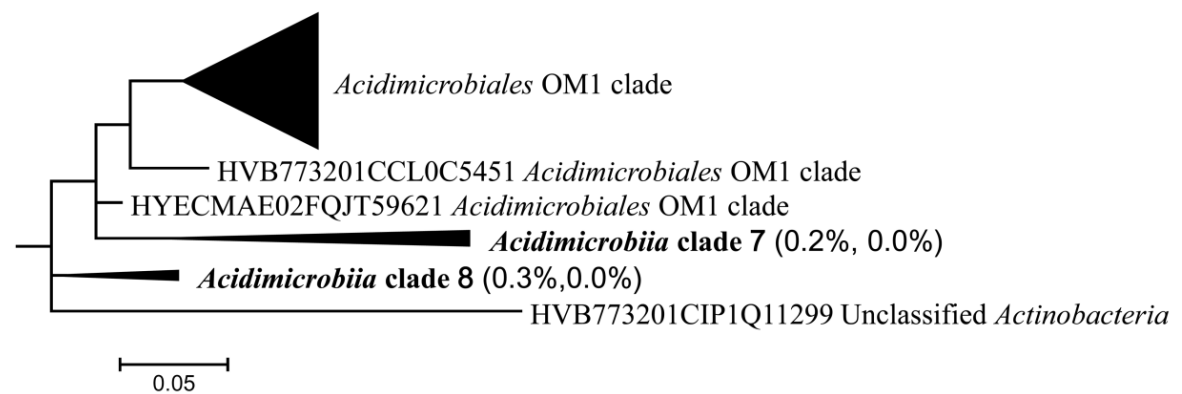

G

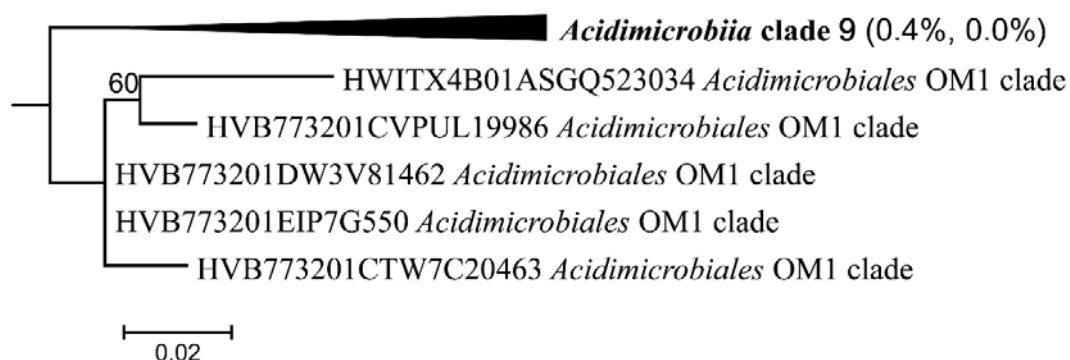

H

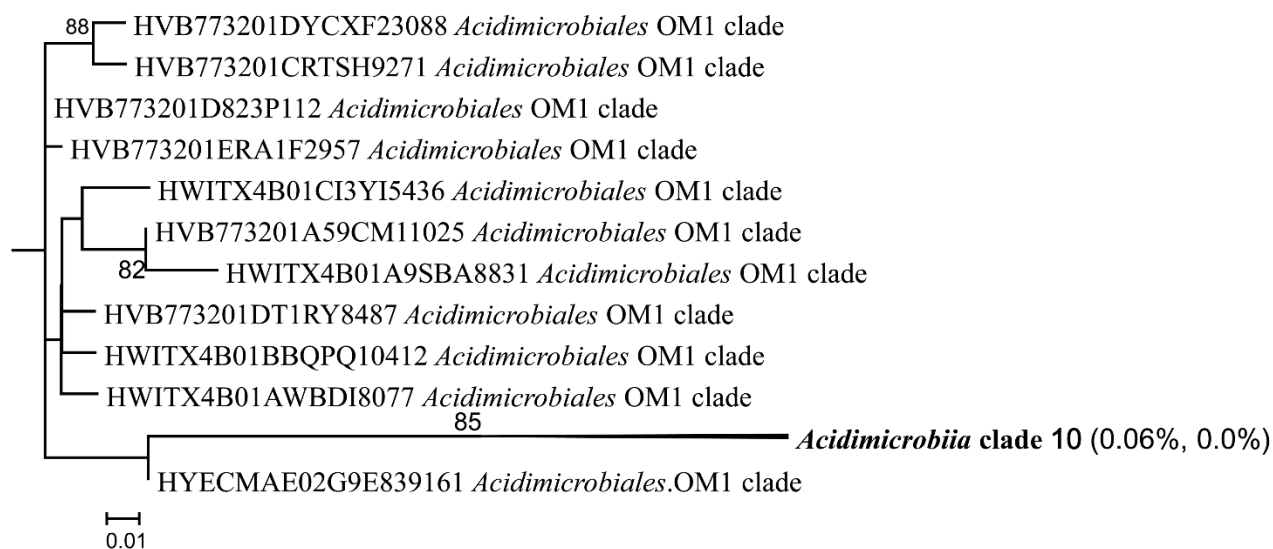

I

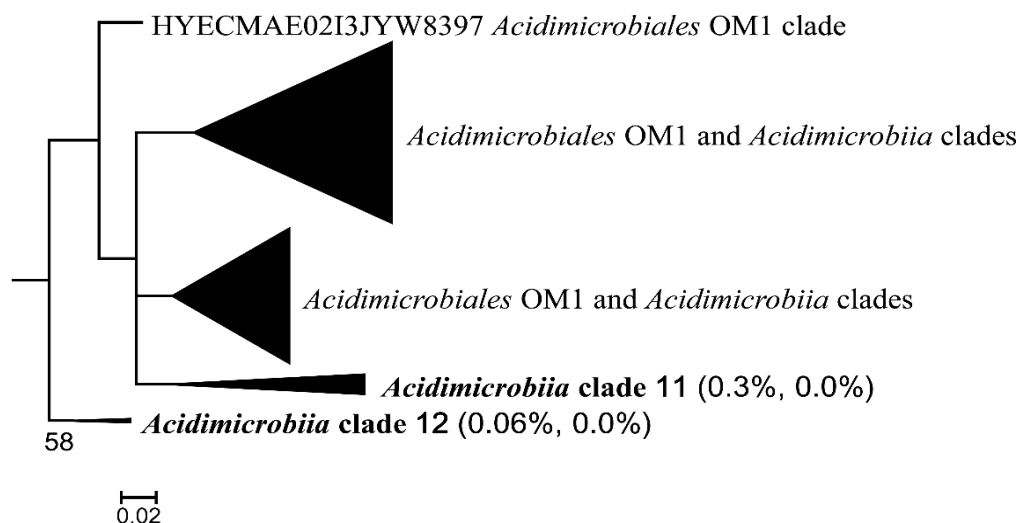

Supplement: Figure S5 — Parts of the zoom-in phylogenetic tree of Figure 7 showing the relationships between the 14 Acidimicrobiia clades and the OM1 clade. Taxonomic assignments of the sequences were based on both the RDP and SILVA databases. Numbers at branch nodes are percentages of bootstrap replicates of 100 resamplings (only values above 50% are shown). Numbers in the parentheses following each clade indicate the relative abundance of pyrosequencing reads and isolates, respectively. Bar = nucleotide changes per site. [file Image5.PDF]
