# Supplementary material for: Diversity, Biogeography, and Biodegradation Potential of Actinobacteria in the Deep-Sea Sediments along the Southwest Indian Ridge
Source: Front Microbiol. 2016 Aug 29;7:1340. doi: 10.3389/fmicb.2016.01340 (PMC5002886; doi:10.3389/fmicb.2016.01340)
Supplement: Table S1 — The geographical distance matrix. [file Table1.DOCX]

### Table S1. The geographical distance matrix^*^.

| Sample ID | M1 | M2 | M3 | M4 | M5 | M6 | M7 | M8 | M9 |
| --- | --- | --- | --- | --- | --- | --- | --- | --- | --- |
| M1 | 0 | 6.872 | 6.756 | 4.986 | 4.423 | 4.359 | 5.523 | 5.558 | 5.576 |
| M2 | 964.92 | 0 | 4.66 | 6.709 | 6.782 | 6.793 | 6.572 | 6.559 | 6.553 |
| M3 | 859.483 | 105.585 | 0 | 6.571 | 6.654 | 6.667 | 6.412 | 6.397 | 6.389 |
| M4 | 146.31 | 819.429 | 713.909 | 0 | 4.147 | 4.289 | 4.652 | 4.739 | 4.777 |
| M5 | 83.367 | 881.642 | 776.183 | 63.219 | 0 | 3.196 | 5.119 | 5.17 | 5.197 |
| M6 | 78.197 | 891.609 | 786.049 | 72.898 | 24.432 | 0 | 5.178 | 5.232 | 5.255 |
| M7 | 250.476 | 714.718 | 609.213 | 104.742 | 167.11 | 177.31 | 0 | 2.495 | 2.7 |
| M8 | 259.281 | 705.649 | 600.202 | 114.324 | 175.997 | 187.152 | 12.121 | 0 | 1.644 |
| M9 | 263.97 | 700.996 | 595.531 | 118.75 | 180.652 | 191.507 | 14.874 | 5.173 | 0 |

^*^Lower triangular, the pairwise geographic distance (km) between the sampling sites; upper triangular, the ln transformed pairwise geographic distance (km) between the sampling sites.
