# Supplementary material for: Diversity, Biogeography, and Biodegradation Potential of Actinobacteria in the Deep-Sea Sediments along the Southwest Indian Ridge
Source: Front Microbiol. 2016 Aug 29;7:1340. doi: 10.3389/fmicb.2016.01340 (PMC5002886; doi:10.3389/fmicb.2016.01340)
Supplement: Table S2 — Summary of the analyses on actinobacterial diversity in shallow and deep seas using culture-dependent and -independent methods. [file Table2.DOCX]

### Table S2. Summary of the analyses on actinobacterial diversity in shallow and deep seas using culture-dependent and -independent methods.

| Sampling site(s) | Habitat(s) | No. of Samples | Depth (m) | Method | Reads/  strains | No. of classes (dominant) | No. of genera/Families (dominant) | Reference |
| --- | --- | --- | --- | --- | --- | --- | --- | --- |
| SWIR | Sediments | 8 | 1662-4000 | Pyrosequen-cing | 2672 | 5 (*Actinobacteria, Acidimicrobiia*) | 44 (*Iamia, Gordonia, Tsukamurella, Microbacterium, Streptomyces*), 26 families | This study |
| SWIR | Water | 1 | 2800 | Pyrosequencing | 573 | 4 (*Actinobacteria, Acidimicrobiia*) | 34 (*Streptomyces, Propionibacterium, Brevibacterium, Tsukamurella, Microbacterium, Arthrobacter, Gordonia, Kitasatospora, Streptacidiphilus*), 21 families | This study |
| SWIR | Sediments | 8 | 1662-4000 | Cultivation | 146 | 1 (*Actinobacteria*) | 28 (*Kocuria, Microbacterium, Rhodococcus, Arthrobacter, Streptomyces, Brevibacterium*) | This study |
| SWIR | Water | 1 | 2800 | Cultivation | 30 | 1 (*Actinobacteria*) | 13 (*Brevibacterium, Kocuria, Micrococcus, Nocardiopsis*) | This study |
| Arctic | Sediments | 2 | 2493.5, 3995 | Clone library | 135 | 2 (*Acidimicrobiia, Actinobacteria*) | 9 (*Ilumatobacter, Pseudonocardia, Microbacterium, Iamia, Janibacter*) | (Zhang et al., 2014) |
| Arctic | Sediments | 2 | 34, 360.7 | Clone library | 210 | 2 (*Acidimicrobiia, Actinobacteria*) | 9 (*Ilumatobacter, Iamia, Pseudonocardia, Demequina*) | (Zhang et al., 2014) |
| Arctic | Sediments | 21 | 34-3995 | Cultivation | 152 | 1 (*Actinobacteria*) | 11 (*Pseudonocardia, Mycobacterium, Brevibacterium, Kocuria, Micrococcus*) | (Zhang et al., 2014) |
| Japan Trench, Canary Basin, and fjords in Norway | Sediments | Not mentioned | 5-6455 | Cultivation | > 800 | 1 (*Actinobacteria*) | 12 genera (Not mentioned) | (Maldonado et al., 2005) |
| Bay of Fundy | Intertidal sediments | 8 | 9-11 | Pyrosequen-cing | 1149 | 1 (*Actinobacteria*) | 14 families (*Nocardioidaceae, Microbacteriaceae, Demequinaceae, Micrococcaceae, Intrasporangiaceae)*) | (Duncan et al., 2014) |
| Bay of Fundy | Intertidal sediments | 8 | 9-11 | Cultivation | 446 | 1 (*Actinobacteria*) | 4 (*Streptomyces*) | (Duncan et al., 2014) |

# Supplementary references

Duncan, K., Haltli, B., Gill, K.A., and Kerr, R.G. (2014). Bioprospecting from marine sediments of New Brunswick, Canada: Exploring the relationship between total bacterial diversity and actinobacteria diversity. *Mar. Drugs.* 12, 899-925.

Maldonado, L.A., Stach, J.E., Pathom-aree, W., Ward, A.C., Bull, A.T., and Goodfellow, M. (2005). Diversity of cultivable actinobacteria in geographically widespread marine sediments. *Antonie van Leeuwenhoek* 87, 11-18. doi: 10.1007/s10482-004-6525-0.

Zhang, G., Cao, T., Ying, J., Yang, Y., and Ma, L. (2014). Diversity and novelty of actinobacteria in Arctic marine sediments. *Antonie van Leeuwenhoek* 105, 743-754. doi: 10.1007/s10482-014-0130-7.
